# Deep learning embedded latent class joint modelling of time-to-event and longitudinal data

**DOI:** 10.1177/09622802261465334

**Published:** 2026-07-10

**Authors:** Tristan John Elers Harris, Najmeh Nakhaei Rad, Sphiwe Bonakele Skhosana

**Affiliations:** 1 Department of Statistics, University of Pretoria, Pretoria, South Africa

**Keywords:** Mixtures of experts, joint modelling, joint latent class model, neural networks, survival analysis, longitudinal modelling

## Abstract

Joint modelling of longitudinal and time-to-event data is a powerful tool for analysing complex medical data. Joint latent class models (JLCMs), in particular, provide additional prognostic insight by clustering subjects into latent subgroups within a mixture of experts framework. Traditionally, the mixing proportions are modelled using a multinomial logistic regression. Recent work in mixture regression has proposed a neural network alternative, implemented through the neural network expectation–maximization algorithm, which offers greater flexibility by capturing nonlinearities that may be missed by logistic regression. In this article, we extend the JLCM by applying the neural network to the modelling the mixing proportions captured in the class-membership submodel. The model estimated using an expectation–maximization-type algorithm. We call this the neural-network JLCM (NN-JLCM). We formally define the method, evaluate its performance through extensive simulations, and apply it to real data. The results show that NN-JLCM improves predictive accuracy and class separation compared with traditional JLCMs, while maintaining competitiveness in terms of overall model fit. The code for the article is available at https://github.com/tharris0924/nn-jlcm_p1.

## 1. Introduction

Longitudinal studies are typically interested in collecting multiple kinds of data on subjects, often a repeated measurement response linked to some event-time of interest, as well as covariates collected at baseline. Examples across multiple disciplines include cognitive assessments recorded repeatedly alongside time to dementia onset or death^1^; recidivism risk scores derived from supervisor evaluations and time until reincarceration^2^; mortgage default times paired with current unpaid balances^3^; longitudinal measures of disability and time to tracheostomy or death in amyotrophic lateral sclerosis trials assessing olesoxime efficacy^4^; and systemic cytokine biomarkers examined in relation to pneumonia progression and risk of severe sepsis or death.^5^

Joint modelling of time-to-event and longitudinal data typically follows one of two methodologies: shared random effects models (SREMs)^6,7^ and joint latent class models (JLCMs).^8–10^ SREMs assume that the relationship between risk of event failure is linked to one or more longitudinal responses directly. This allows practitioners to observe the implicit association between biomarkers and the risk of event-failure. This kind of model is one of the more popular approaches to joint modelling with much research in joint models over the past two decades being centred around SREMs^6,11,12^ with more recent extensions largely considering Bayesian approaches.^13–15^ However, SREMs may be inadequate when the population under study is heterogenous, that is, made up of homogeneous subpopulations that are typically unobserved. In this case, JLCMs provide a suitable modelling framework.^16,17^

The JLCM assumes that the time-to-event and longitudinal responses are linked by the hidden heterogeneity in the population in the form of latent classes. This approach draws inspiration from mixtures of experts modelling^17,18^ where we consider the marginal probabilities of latent classes to be functions of observable baseline covariates, which are then linked to sub-models of the event-time and longitudinal response.^4^ This approach has received much attention in terms of extensions for the longitudinal and survival submodels,^8,16^ but very little research has been done for the class-membership model, which is typically assumed to be a multinomial logistic regression. The exception is Zhang and Simonoff,^17^ who considered a tree-based class-membership model, whereby the decision tree would split variables based on a hypothesis test of conditional independence.

The logistic regression model, while useful, requires the validation of the linear parametric assumptions.^19^ If the relationship between the cluster-membership and the covariates is nonlinear, then the logistic regression cannot adequately capture this relationship. In the mixture modelling literature, there are studies which consider alternate forms of the class-membership model or mixing proportion function. Young and Hunter^20^ considered a nonparametric function of predictors for the mixing proportions, showcasing better performance compared to the standard multinomial logistic regression. Huang and Yao^21^ also considered a semiparametric extension whereby the mixing proportions were governed by a kernel regression technique with a local likelihood. Xue and Yao^19^ proposed an interesting extension by modelling the mixing proportions by way of a neural network combined with maximum likelihood estimation, yielding superior results than either a kernel-based approach or a traditional logistic regression approach. Furthermore, deep learning has also been used in modelling shared parameter joint models with much success in prognostic predictions.^22–24^

The JLCM framework is currently only available in Proust-Lima et al.,^9^ whereby the class-membership model used is a multinomial regression model, with no other readily available competitor.^4^ The model is estimated using a Marquardt algorithm, a Newton-like algorithm, but the approach suffers from flexibility issues, in that the class-membership model cannot be replaced with an approach that has been shown to yield improved classification and prediction. The work of Zhang and Simonoff^17^ is one such approach, where the authors adopted machine learning for JLCMs, which allows practitioners to consider a non-linear and nonparametric approach for the class membership. By using a tree-based approach, this yielded improved predictive capabilities for the survival and longitudinal model called the joint latent class tree (JLCT). This approach is considered a two-stage modelling approach, where the tree is fit using recursive binary splitting using a hypothesis test as the splitting criterion (similar in fashion to survival trees), and the longitudinal and survival models are fitted separately using Newton-Raphson approaches. However, there is no such unified approach that combines machine learning and maximum likelihood in the estimation of JLCMs to take advantage of the benefits of a hybridized approach.

This article proposes a novel approach for estimating the mixing proportions in a JLCM by using a neural network. An expectation–maximization (EM) algorithm^25^ is developed to estimate the model parameters. The advantage of the approach allows practitioners to flexibly model nonlinear relationships between class-membership and the covariates, without relying on parametric assumptions of logistic regressions. This is to say that machine-learning adapted approaches within JLCMs can yield superior clustering solutions and prognostic predictions. These advantages are confirmed by our extensive simulation results and application. As a result, better prognostic predictions of survival and longitudinal outcomes are possible using the neural network for the class-membership. This, in turn, leads to more robust clustering solutions in the face of increasingly complex disease modelling.

The rest of the article is organized as follows. Section 2 considers the materials and methods needed for the study by giving a background on joint modelling, JLCM and our proposed method using deep learning. Section 3 details an extensive simulation study where the proposed neural-network JLCM (NN-JLCM) consistently outperforms the traditional JLCM across three simulation studies. Additional tables and simulation results are given in the Supplemental Material. In Section 4, NN-JLCM is compared with JLCM, the JLCT of Zhang and Simonoff,^17^ and SREM of Rizopoulos^26^ across additional experiments to further assess the finite sample properties of the NN-JLCM method. The first such experiment is where all the simulations are repeated from Section 3, but now an array of models is fitted with varying chosen latent classes. We report on the latent class selection criteria to determine the model’s efficacy at selecting the correct number of latent classes. The second experiment considers fitting the models to a dataset with considerable cluster overlap. The final experiment considers a double-fold evaluation of the assumptions of the NN-JLCM, where in the first instance, we generate from an SREM and fit the NN-JLCM to the data, and in the second instance, we generate from a JLCM and fit the NN-JLCM to the data. This is to evaluate the robustness of the NN-JLCM in the face of model misspecification. Section 5 considers an application of the model to the PAQUID dataset,^27^ where the longitudinal measure we consider is the normalized mini-mental state examination (MMSE) and the event-time we consider is dementia. We show that NN-JLCM has superior predictive capabilities for both the longitudinal and survival outcomes, while also yielding better class separation compared to the JLCM, JLCT, and SREM. In Section 6, after discussing the results obtained, we conclude the article and provide direction for future research.

## 2. Materials and methods

### 2.1. A brief primer on time-to-event and repeated measurement analysis

Let the random variable $T_{i}=\min(T_{i}^{*},C_{i})$ be the actual survival time, where $T_{i}^{*}$ is the true survival time and $C_{i}$ is the censored time, with censoring indicator,

$$\begin{array}{r} \delta_{i}=I(T_{i}^{*}\leq C_{i})=\left\{ \begin{array}{ll} 1 & \text{if an event is uncensored for the}\;i\text{th subject}\\ 0 & \text{if an event time is right-censored for the}\;i\text{th subject} \end{array}\right. \end{array}$$

Then, the observed data for each subject i=1,…,n , consist of the tuples $\left(t_{i},\delta_{i}\right)$ . A traditional proportional hazards model takes the form,^28^

(1) $$h_{i}\left(t_{i}|\vartheta ,\zeta \right)=h_{0}(t|\zeta )\exp\;\left(w_{i}^{⊺}\vartheta \right)$$

where $h_{0}(t)$ is the baseline hazard function with parameters ζ , ϑ is a p -vector of baseline regression parameters with associated p -vector of baseline covariates $w_{i}^{⊺}$ . Therefore, the survival function is given by the following equations:

(2) $$\begin{array}{rl} S\left(t|\vartheta ,\zeta \right) & =\exp\;\left(-\int_{0}^{t}h_{0}(s|\zeta )\exp\;\left\{w_{i}^{⊺}\vartheta \right\}ds\right) \end{array}$$

(3) $$\begin{array}{rl} & =\exp\;\left(-H\left(t|\vartheta ,\zeta \right)\right) \end{array}$$

In addition to survival times, there might be other measurements made on the subject over time. Let $y_{ij}$ be the repeated measurement of interest recorded for time point $t_{ij}$ at $j=1,\ldots ,n_{i}$ occasions, for a sample of i=1,…,n subjects, so that $y_{i}=(y_{i1},\ldots ,y_{in_{i}}{)}^{⊺}$ . The standard linear mixed-effects model is given by the following equations^29,30^:

(4) $$\begin{array}{rl} & y_{ij}=x_{ij}^{⊺}\beta +z_{ij}^{⊺}b_{i}+\varepsilon_{ij},\;j=1,\ldots ,n_{i},\;\;i=1,2,\ldots ,n \end{array}$$

$$\begin{array}{rl} b_{i} & =(b_{1i},b_{2i},\ldots ,b_{qi}{)}^{⊺}∼N_{q}(0,D),\;\varepsilon_{i}=(\varepsilon_{i1},\varepsilon_{i2},\ldots ,\varepsilon_{in_{i}})∼N_{p}\left(0,R_{i}\right) \end{array}$$

where $\beta =(\beta_{1},\beta_{2},\ldots ,\beta_{p}{)}^{⊺}$ is the p -vector of fixed effects parameters, $b_{i}$ denotes the q -dimensional vector of random-effect parameters for the ith subject, $x_{ij}=(x_{ij1},x_{ij2},\ldots ,x_{ijp}{)}^{⊺}$ is the p -dimensional vector for the ith subject’s covariates at time $t_{ij}$ , $z_{ij}=(z_{ij1},z_{ij2},\ldots ,z_{ijq}{)}^{⊺}$ is the q -dimensional vector of the random effect covariates, and $b_{i}$ is independent of the $n_{i}$ -dimensional vector of the error term $\varepsilon_{i}.$ Typically, we assume that D is an unstructured covariance matrix for the random-effects. For the covariance matrix of the errors, we assume $R_{i}=\sigma^{2}I_{n_{i}}$ , where $I_{n_{i}}$ is an $n_{i}$ -dimensional identity matrix.

### 2.2. Joint latent class model

Unlike the SREMs, the JLCM does not directly link the time-to-event model and linear mixed-effects model with an association parameter. Instead, the assumption is that the association between the time-to-event and longitudinal processes is captured by the predicted latent classes.^9,10^ Every latent class has a class-specific repeated measurement trajectory and a class-specific hazard function. The JLCM draws inspiration from finite mixture modelling, specifically, finite mixtures of experts.^18^

Fundamentally, it is also assumed that the repeated measurements and the time-to-event are independent conditionally on the latent class. The reasoning for this: it is conjectured that the observed association between the time-to-event and longitudinal data over the entire population is spurious, largely driven by the heterogeneity in levels of the variables between latent classes.^17^

Let the n subjects under study be divided into a finite number of G homogenous latent groups. The class belonging for the ith subject is denoted by a categorical variable $c_{i}$ . The probability of latent class membership is denoted by the following multinomial regression model according to the covariates $v_{i}^{⊺}$ ,^16^

(5) $$\pi_{ig}=\mathrm{Pr}\left(c_{i}=g|V=v\right)=\frac{\exp\;\left(v_{i}^{⊺}\xi_{g}\right)}{\sum_{l=1}^{G}\exp\;\left(v_{i}^{⊺}\xi_{l}\right)},\;g=1,2,\ldots ,G$$

where $\xi_{g}=(\xi_{1g},\xi_{2g},\ldots ,\xi_{dg}{)}^{⊺}$ denotes the class-specific parameters and $\xi_{G}=0$ for identifiability.

The class-specific linear mixed-effects model is given as follows:

(6) $${y_{ij}|}_{c_{i}=g}=x_{ij}^{⊺}\beta_{g}+{\tilde{x}}_{ij}^{⊺}\tilde{\beta }+z_{ij}^{⊺}b_{ig}+\varepsilon_{ij}$$

for $j=1,\ldots ,n_{i},$ measurements and i=1,2,…,n subjects. The fixed effects parameters are now split among class-specific effects, $\beta_{g}=(\beta_{1g},\beta_{2g},\ldots ,\beta_{pg}{)}^{⊺}$ with design vector $x_{ij}^{⊺}$ and common effects $\tilde{\beta }=({\tilde{\beta }}_{1},{\tilde{\beta }}_{2},\ldots ,{\tilde{\beta }}_{p}{)}^{⊺}$ with design vector ${\tilde{x}}_{ij}^{⊺}$ . We assume that $x_{ij}^{⊺}$ and ${\tilde{x}}_{ij}^{⊺}$ do not intersect for identifiability purposes.^4^

The q -vector $b_{ig}=(b_{1ig},b_{2ig},\ldots ,b_{qig}{)}^{⊺}$ denotes the class-specific random-effects with associated design vector $z_{i}^{⊺}$ and class-specific distribution $b_{i}{|}_{c_{i}=g}∼N(\mu_{g},D_{g})$ , which can be rewritten as a mixture model, $b_{i}∼\sum_{g=1}^{G}\pi_{ig}N_{q}(\mu_{g},D_{g})$ . We assume that $b_{ig}$ are independent over classes and subjects. The variance-covariance matrix $D_{g}$ of the random-effects can be parameterized to be class-specific or common over classes. If class-specific, then $D_{g}$ = $\omega_{g}^{2}D$ , where $\omega_{g}^{2}$ represents a proportional coefficient for each g , such that the variance-covariance matrices are proportional to each other and $\omega_{G}^{2}=1$ for identifiability. The $n_{i}$ -vector $\varepsilon_{i}=(\varepsilon_{i1},\varepsilon_{i2},\ldots ,\varepsilon_{in_{i}}{)}^{⊺}$ denotes the residuals with associated distribution $\varepsilon_{i}∼N_{p}(0,R_{i}).$ The residual variance-covariance matrix is assumed typically to be diagonal $R_{i}=\sigma^{2}I_{n_{i}}$ .

The class-specific hazards function is given by the following equation:

(7) $$h_{i}(t|c_{i}=g)=h_{0g}(t|\zeta_{g})\exp\;\left(w_{i}^{⊺}\vartheta_{g}+{\tilde{w}}_{i}\vartheta \right)$$

where $\zeta_{g}$ denotes the class-specific baseline-hazard parameters, and $\vartheta_{g}$ denotes the class-specific hazard ratios with associated covariates $w_{i}^{⊺}$ and ϑ denotes the hazard ratios common over all classes. The baseline hazard function can be parameterized as an array of parametric functions such as the two-parameter Weibull, whereby $h_{0g}(t|\zeta_{g})=\zeta_{1g}\zeta_{2g}(t\zeta_{1g}{)}^{\zeta_{1g}-1}$ , or semiparametric functions such as piecewise functions or spline functions.

Let $\Theta_{G}$ denote the complete parameter vector for a fixed number of classes G , and let $\theta_{g}$ denote the class-specific vector of parameters for the gth class. Then, let $f(y_{i}|\theta_{g})$ denote the contribution of the likelihood function associated with the repeated measurements, conditional on class, and let $S_{i}(t|\theta_{g})$ denote the class-specific survival function. Therefore, the full observed log-likelihood function is

(8) $$L(\Theta_{G})=\sum_{i=1}^{n}\log\;\sum_{g=1}^{G}\pi_{ig}f(y_{i}|\theta_{g})h_{i}(t|\theta_{g}{)}^{\delta_{i}}S_{i}(t|\theta_{g})$$

The likelihood in equation (8) is maximized using an extended Marquardt algorithm, a Newton-type algorithm with stringent convergence criteria.^9,31^ In the extended Marquardt algorithm, the parameter vector $\theta_{G}$ is iteratively updated until convergence according to the following equation:

(9) $$\Theta_{G}^{\left(l+1\right)}=\Theta_{G}^{\left(l\right)}-\delta^{*}{\tilde{H}}^{{\left(l\right)}^{-1}}\frac{\partial L(\Theta_{G}^{\left(l\right)})}{\partial \Theta_{G}^{\left(l\right)}}$$

where $L(\Theta_{G}^{\left(l\right)})$ and $\frac{\partial L(\Theta_{G}^{\left(l\right)})}{\partial \Theta_{G}^{\left(l\right)}}$ is the log-likelihood and associated gradient at iteration l , and $\delta^{*}$ (equal to 1 by default) is internally adjusted to ensure that each update improves the log-likelihood. The matrix $\tilde{H}$ represents a diagonal-inflated version of the Hessian, used to maintain positive-definiteness. When required, its diagonal entries are modified as follows:

(10) $${\tilde{H}}_{ii}=H_{ii}+\lambda^{*}[(1-\eta )|H_{ii}|+\eta \;\text{tr}(H)]$$

where H is the Hessian matrix with diagonal elements $H_{ii}$ , and $\lambda^{*}$ and η are tuning parameters. Initially, $\lambda^{*}$ and η are set to 0.01 and are adaptively adjusted at each iteration—reduced when $\tilde{H}$ is positive-definite and increased otherwise. The gradient of the log-likelihood is approximated using finite differences, whereby the first derivatives are obtained by central differences and the second derivatives are computed by forward differences.

Convergence is declared when three conditions are simultaneously satisfied. The first is for parameter stability, the second is for log-likelihood stability, and the last one is a derivative-based criterion for the relative distance to the maximum. The variance-covariance matrix of the estimates, denoted $\hat{\mathrm{Var}}({\hat{\theta }}_{G})$ , is estimated from the inverse of the Hessian matrix. To begin the estimation process, we use the asymptotic normality properties of $\Theta_{G}$ by drawing from the maximum likelihood estimates (MLEs) of a single-cluster model ( $\theta ∼N({\hat{\theta }}_{g=1},\hat{\mathrm{Var}}({\hat{\theta }}_{g=1}))$ ) as initial values.

Classification with the model is performed using the posterior conditional probabilities, or responsibilities, which are computed as follows:

(11) $$\gamma_{ig}=\mathrm{Pr}(c_{i}=g|Y_{i},T_{i},\delta_{i},\theta_{g})=\frac{\pi_{ig}f(y_{i}|\theta_{g})h_{i}(t|\theta_{g}{)}^{\delta_{i}}S_{i}(t|\theta_{g})}{\sum_{g=1}^{G}\pi_{ig}f(y_{i}|\theta_{g})h_{i}(t|\theta_{g}{)}^{\delta_{i}}S_{i}(t|\theta_{g})}$$

Using equation (11), we can determine the model-based clusterings based on the predicted responsibilities, as follows:

$$\hat{c_{i}}=\underset{g}{\mathrm{argmax}}\;{\hat{\gamma }}_{ig}$$

for i=1,…,n and g=1,…,G . Using this, we can compute the individual predicted survival (where ${\hat{S}}_{ig}(t)$ denotes the predicted survival probabilities for subject i and group g ) and longitudinal curves (where ${\hat{y}}_{ijg}$ denotes the predicted response for subject i at recording j in group g ) as the weighted sum of the responsibilities and the class specific predictions as follows:

(12) $${\hat{S}}_{i}(t)=\sum_{g=1}^{G}{\hat{\gamma }}_{ig}{\hat{S}}_{ig}(t),\;{\hat{y}}_{ij}=\sum_{g=1}^{G}{\hat{\gamma }}_{ig}{\hat{y}}_{ijg}$$

Finally, the JLCM assumes that the latent class structure fully captures the dependency between the longitudinal marker(s) and the time-to-event. Several methods can be used to evaluate this assumption, including posterior classification analysis, residual analysis, and a score test.^4^ In the score test, the alternative hypothesis $H_{1}$ allows for an additional dependence between the longitudinal random-effects and the time-to-event beyond the latent classes

(13) $$h_{i}(t|\theta_{g})=h_{0g}(t|\zeta_{g})\exp\;\left(w_{i}^{⊺}\vartheta_{g}+{\tilde{w}}_{i}^{⊺}\vartheta +b_{ig}^{⊤}\eta \right)$$

where η links the p random-effects $b_{ig}$ from the longitudinal submodel to the survival submodel.

Testing conditional independence reduces to $H_{0}:\eta =0$ (vs. $H_{1}:\eta \neq 0$ ). The corresponding score statistic is

(14) $$U=\sum_{i=1}^{N}\sum_{g=1}^{G}{\hat{\gamma }}_{ig}\left(\delta_{i}-H_{ig}(t_{i})\right){\hat{b}}_{ig}$$

where ${\hat{b}}_{ig}$ is the empirical Bayes estimate in class g , $H_{ig}(t_{i})$ is the class-specific cumulative hazard, and η is the q -vector of parameters linking the random effects to the survival model. Under $H_{0}$ , the standardized statistic $U^{⊤}\hat{\mathrm{Var}}(U{)}^{-1}U$ follows a $\chi^{2}$ distribution with q degrees of freedom. Simulation studies showed the score test to be more powerful than alternative methods for detecting violations of the conditional independence assumption.^10,32^

### 2.3. Neural network JLCMs

Here we consider our proposed model. The model parameterizations for the longitudinal and survival submodels remain the same. In this article, we consider a neural network to model the mixing proportions $\pi_{ig}$ , as opposed to the logistic model in the literature, by proposing an EM algorithm. We first explain the architecture of the neural network, and second, we provide the details of the EM algorithm for how this new class of joint models is implemented.

#### 2.3.1. Neural networks

Neural networks are inspired by the way in which information travels and is stored in the human brain. This is where *neurons*, the most basic processing unit of the brain, would ‘fire’ by sending electrical impulses along neural pathways to connect to other neurons through synaptic links (or synapses).

There are approximately 90 billion neurons and 100 trillion synapses in the human brain, where each neuron is connected through several thousand synapses. As a neuron receives information from another neuron, it can be induced to fire as well, through signals known as neurotransmitters, which are released at the synapses. Certain synaptic pathways inhibit or exacerbate the neural impulse so that firing an input neuron will reduce the likelihood of the output neuron firing.^33^

Mathematical properties of neural networks were first pioneered by McCulloch and Pitts^34^ with the first simplistic neural network models such as the *perceptron* studied in the seminal work of Rosenblatt.^35^ Mathematically, the output of the gth output neuron in a two-layer perceptron can be represented as follows:

(15) $$f_{g}(x_{i}|\varpi ,\alpha )=\sigma_{g}(\alpha_{g}^{\left(2\right)}+\sum_{l=1}^{L}\varpi_{lg}^{\left(2\right)}\sigma_{l}(\alpha_{l}^{\left(1\right)}+\sum_{j=1}^{p}x_{ij}\varpi_{jl}^{\left(1\right)}))$$

where $x_{i1},\ldots ,x_{ip}$ represents the j=1,…,p features of the i th training example and $\varpi_{il}^{\left(1\right)}$ are weights of the lth neuron for the jth feature input in the first layer, and $\alpha_{l}$ is the bias of the lth neuron. The convex sum of the weights and neuron inputs plus the bias yields quantity $a_{g}$ , which is known as the *pre-activation*. This represents the strength of the synaptic links. The pre-activation is fed into a nonlinear *activation function* f(⋅) which gives an output called the *activation*. We consider the softmax function for the activation functions in the neural network, so that the activation function for the gth neuron

(16) $$\sigma_{g}(a_{g})=\frac{1}{1+\exp\;(-a_{g})}$$

**Figure 1.**
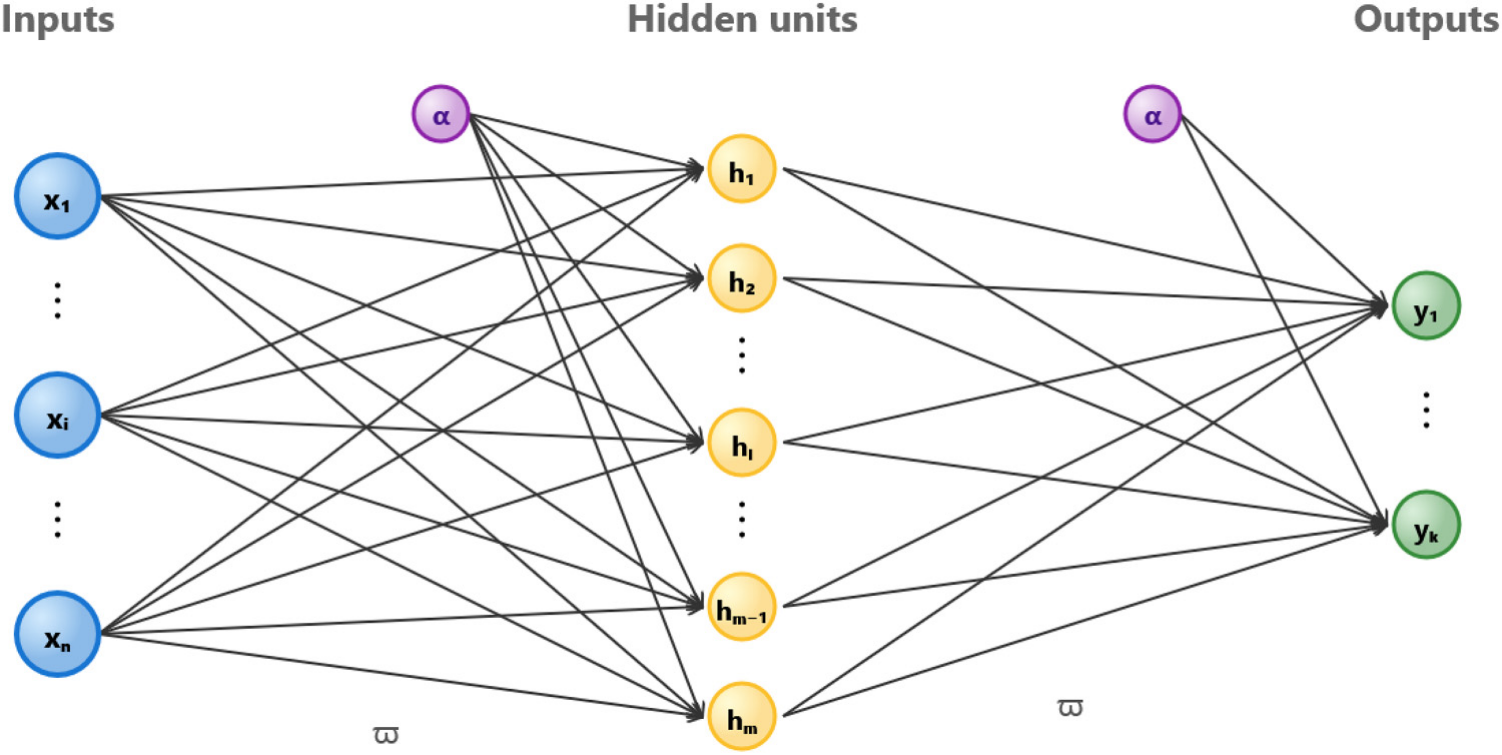
Architecture for a single hidden-layer neural network.

The architecture of this two-layer neural network is shown in Figure 1. Naturally, this model can be extended to more than two layers.

Estimation of the weights and biases is done using a variant of gradient descent and back-propagation, which seeks to minimize the following function:

(17) $$\mathrm{CE}(\varpi ,\alpha |y,X)=-\sum_{i=1}^{n}\sum_{g=1}^{G}y_{ig}\log\;f_{g}(x_{i}|\varpi ,\alpha )$$

where $y=(y_{1},y_{2},\ldots ,y_{n})$ are the targets with each $y_{i}$ having a one-of-K coding for the true k th class and 0s otherwise, and $x=(x_{1},x_{2},\ldots ,x_{n})$ with $x_{i}=(x_{i1},\ldots ,x_{ip})$ .

#### 2.3.2. Backpropagation and gradient descent

Backpropagation is an algorithm that computes gradients of the loss function with respect to the network parameters (weights ϖ and biases α ) using the chain rule of calculus. This approach is used in tandem with gradient descent. The algorithm begins with a forward pass (forward-propagation) where for each training example i , compute the pre-activations $a_{l}$ and activations $h_{l}$ of the hidden layer,

(18) $$\begin{array}{rl} a_{l}^{\left(1\right)} & =\alpha_{l}^{\left(1\right)}+\sum_{j=1}^{p}x_{ij}\varpi_{jl}^{\left(1\right)}\;\text{for }l=1,\ldots ,L \end{array}$$

(19) $$\begin{array}{rl} h_{l} & =\sigma_{l}(a_{l}^{\left(1\right)})=\frac{1}{1+\exp\;(-a_{l}^{\left(1\right)})} \end{array}$$

and compute the pre-activations $a_{g}$ and activations $f_{g}$ of the output layer

(20) $$\begin{array}{rl} a_{g}^{\left(2\right)} & =\alpha_{g}^{\left(2\right)}+\sum_{l=1}^{L}\varpi_{lg}^{\left(2\right)}h_{l}\;\text{for }g=1,\ldots ,G \end{array}$$

(21) $$\begin{array}{rl} f_{g} & =\sigma_{g}(a_{g}^{\left(2\right)})=\frac{1}{1+\exp\;(-a_{g}^{\left(2\right)})} \end{array}$$

Then, using the chain rule, we compute gradients layer-by-layer, moving backwards from output to input in the backward pass. For the output layer, the error signal at the output layer is given by the following equation:

(22) $$\delta_{g}^{\left(2\right)}=\frac{\partial \mathrm{CE}}{\partial a_{g}^{\left(2\right)}}=f_{g}-y_{ig}$$

This follows from the derivative of cross-entropy with respect to the sigmoid pre-activation. The gradients for output layer parameters are given as follows:

(23) $$\begin{array}{rl} \frac{\partial \mathrm{CE}}{\partial \varpi_{lg}^{\left(2\right)}} & =\delta_{g}^{\left(2\right)}h_{l} \end{array}$$

(24) $$\begin{array}{rl} \frac{\partial \mathrm{CE}}{\partial \alpha_{g}^{\left(2\right)}} & =\delta_{g}^{\left(2\right)} \end{array}$$

The error signal for the hidden layer is given as follows:

(25) $$\delta_{l}^{\left(1\right)}=\frac{\partial \mathrm{CE}}{\partial a_{l}^{\left(1\right)}}=\left(\sum_{g=1}^{G}\delta_{g}^{\left(2\right)}\varpi_{lg}^{\left(2\right)}\right)\cdot \sigma_{l}^{\prime }(a_{l}^{\left(1\right)})$$

where $\sigma_{l}^{\prime }(a_{l}^{\left(1\right)})=\sigma_{l}(a_{l}^{\left(1\right)})(1-\sigma_{l}(a_{l}^{\left(1\right)}))=h_{l}(1-h_{l})$ is the derivative of the sigmoid function. Equation (25) shows us that the error signals for a particular hidden unit are computed by propagating the error signals backwards from units higher up in the network. The gradients for hidden layer parameters are then given as follows:

(26) $$\begin{array}{rl} \frac{\partial \mathrm{CE}}{\partial \varpi_{jl}^{\left(1\right)}} & =\delta_{l}^{\left(1\right)}x_{ij} \end{array}$$

(27) $$\begin{array}{rl} \frac{\partial \mathrm{CE}}{\partial \alpha_{l}^{\left(1\right)}} & =\delta_{l}^{\left(1\right)} \end{array}$$

Finally, the parameters can be updated by way of gradient descent (or other variants)

(28) $$\begin{array}{rl} \varpi^{\left(m\right)} & \leftarrow \varpi^{\left(m\right)}-\nu \frac{\partial \mathrm{CE}}{\partial \varpi^{\left(m\right)}} \end{array}$$

(29) $$\begin{array}{rl} \alpha^{\left(m\right)} & \leftarrow \alpha^{\left(m\right)}-\nu \frac{\partial \mathrm{CE}}{\partial \alpha^{\left(m\right)}} \end{array}$$

where ν is the learning rate and m∈{1,2} denotes the layer. The elegance of backpropagation lies in the recursive computation of $\delta^{\left(m\right)}$ values. Each layer’s error signal is computed from the subsequent layer’s error signal, weighted by the connections between them, and modulated by the local activation function derivative. This allows efficient computation of all gradients faster than naive approaches without backpropagation.

#### 2.3.3. Neural network embedded JLCM

The NN-JLCM is our proposed model, and we use an EM algorithm to estimate the parameters of the model. The structures of the longitudinal submodel in equation (6) and the survival submodel in equation (7) remain the same. We begin by briefly describing the EM algorithm and then giving the complete data log-likelihood, and then proceed to the E-steps and M-steps, respectively.

#### 2.3.4. Brief description of the EM algorithm

The EM algorithm, first introduced by Dempster et al.,^25^ is a highly versatile iterative algorithm developed to estimate the maximum likelihood in the context of incomplete data problems. This can include both natural incomplete data problems, where we have censoring, for example, or an artificial incomplete data problem, such as the unknown latent class in finite mixture models. The problem is usually solved by introducing the missing data as a latent variable, resulting in complete data. The logic is that the log-likelihood of the complete data is typically easier to maximize, often in a closed form. The algorithm has two steps: the expectation (E-), where we calculate the expected value of the complete data log-likelihood. The second step of the EM algorithm is to maximize (M-) the expected complete-data log-likelihood function obtained in the E-step. The E- and M-steps are repeated until convergence. A very brief description of how it works follows.

Assuming that we have our complete data matrix represented by X , which can be decomposed into an observed part $X^{o}$ , which is a matrix and a missing part, $z^{m}$ , which is a vector. The objective is to estimate the parameters θ for the complete data model, with only observed information. In the E-step, we compute the expected value of the complete data log-likelihood. The expected value on the auxiliary function Q(⋅) is conditional on the observed data $X^{o}$ and the kth iteration’s estimate of the parameters, $\theta^{\left(k\right)}$

(30) $$\begin{array}{rl} Q\left(\theta |\theta^{\left(k\right)}\right) & =E_{z|X^{o},\theta^{\left(k\right)}}\left[\ln\;f(X|\theta )\right]\\ & =\int \ln\;f\left(X^{o},z^{m}|\theta \right)f\left(z^{m}|X^{o},\theta^{\left(k\right)}\right)dz^{m} \end{array}$$

We then update the parameters in the M-step by the following equation:

(31) $$\theta^{\left(k+1\right)}=\underset{\theta }{\arg\;\max}\;\;Q\left(\theta |\theta^{\left(k\right)}\right)$$

With each successive iteration, it has been shown that the EM algorithm increases the log-likelihood. However, the algorithm does not necessarily converge to a global maximum, but under mild conditions (see Dempster et al.^25^), it always finds a local maximum. A particularly great advantage of the EM algorithm is its numerical stability; however, it is sensitive to initial values and can have a slow rate of convergence.^36^ More details on the EM algorithm can be found by McLachlan and Krishnan^37^ and Murphy.^36,38^

#### 2.3.5. Neural network EM (NNEM) algorithm for NN-JLCM

We now define the NNEM algorithm for estimating the NN-JLCM. As equation (8) is the observed log-likelihood of the JLCM, the complete data log-likelihood can be written using the notation established. Let the unobserved indicator $c_{ig}$ so that $c_{ig}=I(c_{i}=g)$ , where $c_{i}$ is the component label of the ith observation, where g∈{1,…,G} .

Additionally, let the observed covariates comprising the design matrices for the longitudinal, survival, and class-membership submodels be denoted as $D=(D_{\text{long}},D_{\text{surv}},D_{\text{class}})$ , where

-
$D_{\text{long}}=(X,\tilde{X},Z)$ contains the design matrices for the class-specific fixed effects $X=(x_{1},x_{2},\ldots ,x_{n})$ , common fixed effects $\tilde{X}=({\tilde{x}}_{1},{\tilde{x}}_{2},\ldots ,{\tilde{x}}_{n})$ , and random effects $Z=(z_{1},z_{2},\ldots ,z_{n})$ in the longitudinal submodel.
-
$D_{\text{surv}}=(W,\tilde{W})$ contains the design matrices for the class-specific covariates $W=(w_{1},w_{2},\ldots ,w_{n})$ and common covariates $\tilde{W}=({\tilde{w}}_{1},{\tilde{w}}_{2},\ldots ,{\tilde{w}}_{n})$ in the survival submodel.
-
$D_{\text{class}}=V=(v_{1},v_{2},\ldots ,v_{n})$ contains the covariate vectors for the class-membership model.

Let $y=(y_{1},y_{2},\ldots ,y_{n})$ denote the vector of longitudinal responses, $t=(t_{1},t_{2},\ldots ,t_{n})$ denote the survival times vector, and $\delta =(\delta_{1},\delta_{2},\ldots ,\delta_{n})$ denote the vector of event indicators. The complete-data log-likelihood is

(32) $$\ell^{c}(\Theta_{G}|D,y,t,\delta ,c)=\sum_{i=1}^{n}\sum_{g=1}^{G}c_{ig}\log\;(\pi_{g}(v_{i})f(y_{i}|D_{\text{long}},\theta_{g})h_{i}(t_{i}|D_{\text{surv}},\theta_{g}{)}^{\delta_{i}}S_{i}(t_{i}|D_{\text{surv}},\theta_{g}))$$

It then follows that the conditional expectation of equation (32) given the m−1 parameter estimates and the observed data D is computed as follows:

(33) $$\begin{array}{rl} Q\left(\theta ,\theta^{\left(m\right)}\right) & ≜E_{c_{i}|y_{i},t_{i},\delta_{i},D,\theta^{\left(m\right)}}\left[\sum_{i=1}^{n}\log\;p\left(y_{i},t_{i},\delta_{i},c_{i}|D,\theta \right)\right]\\ & =\sum_{i=1}^{n}E_{c_{i}|y_{i},t_{i},\delta_{i},D,\theta^{\left(m\right)}}[\log\;[\prod_{g=1}^{G}(\pi_{g}(v_{i})f(y_{i}|D_{\text{long}},\theta_{g})h_{i}(t_{i}|D_{\text{surv}},\theta_{g}{)}^{\delta_{i}}S_{i}(t_{i}|D_{\text{surv}},\theta_{g}){)}^{I\left(c_{i}=g\right)}]]\\ & =\sum_{i=1}^{n}\sum_{g=1}^{G}E_{c_{i}|y_{i},t_{i},\delta_{i},D,\theta^{\left(m\right)}}\left[I\left(c_{i}=g\right)\right]\log\;[\pi_{g}(v_{i})f(y_{i}|D_{\text{long}},\theta_{g})h_{i}(t_{i}|D_{\text{surv}},\theta_{g}{)}^{\delta_{i}}S_{i}(t_{i}|D_{\text{surv}},\theta_{g})]\\ & =\sum_{i=1}^{n}\sum_{g=1}^{G}\mathrm{Pr}(c_{i}=g|y_{i},t_{i},\delta_{i},D,\theta^{\left(m-1\right)})\log\;[\pi_{g}(v_{i})f(y_{i}|D_{\text{long}},\theta_{g})h_{i}(t_{i}|D_{\text{surv}},\theta_{g}{)}^{\delta_{i}}S_{i}(t_{i}|D_{\text{surv}},\theta_{g})]\\ & =\sum_{i=1}^{n}\sum_{g=1}^{G}\gamma_{ig}^{\left(m\right)}\log\;\pi_{g}(v_{i})+\sum_{i=1}^{n}\sum_{g=1}^{G}\gamma_{ig}^{\left(m\right)}\log\;f(y_{i}|D_{\text{long}},\theta_{g})+\sum_{i=1}^{n}\sum_{g=1}^{G}\gamma_{ig}^{\left(m\right)}\log\;h_{i}(t_{i}|D_{\text{surv}},\theta_{g}{)}^{\delta_{i}}S_{i}(t_{i}|D_{\text{surv}},\theta_{g}) \end{array}$$

where

(34) $$\begin{array}{rl} \gamma_{ig}^{\left(m\right)} & =\mathrm{Pr}(c_{i}=g|y_{i},t_{i},\delta_{i},D,\theta^{\left(m-1\right)})\\ & =\frac{\pi_{g}^{\left(m-1\right)}(v_{i})\;f(y_{i}|D_{\text{long}},\theta_{g}^{\left(m-1\right)})\;h_{i}(t_{i}|D_{\text{surv}},\theta_{g}^{\left(m-1\right)}{)}^{\delta_{i}}\;S_{i}(t_{i}|D_{\text{surv}},\theta_{g}^{\left(m-1\right)})}{\sum_{g=1}^{G}\pi_{g}^{\left(m-1\right)}(v_{i})\;f(y_{i}|D_{\text{long}},\theta_{k}^{\left(m-1\right)})\;h_{i}(t_{i}|D_{\text{surv}},\theta_{k}^{\left(m-1\right)}{)}^{\delta_{i}}\;S_{i}(t_{i}|D_{\text{surv}},\theta_{k}^{\left(m-1\right)})} \end{array}$$

is the posterior probability that subject i belongs to latent class g given all observations, known as the responsibilities.

To update the mixing proportions $\pi_{g}(v_{i})$ , for g=1,2,…,G , we make use of the neural network

(35) $$\pi_{g}\left(v_{i}\right)=f_{g}\left(v_{i}|\varpi ,\alpha \right)$$

We take $\gamma^{\left(m\right)}=\{\gamma_{ig}^{\left(m\right)}:i=1,2,\ldots ,n;g=1,2,\ldots ,G\}$ as the output response variables and $D_{\text{class}}$ as the input covariates and we minimize

(36) $$\mathrm{CE}\left(\varpi ,\alpha |D_{\text{class}},\gamma^{\left(m\right)}\right)=-\sum_{i=1}^{n}\sum_{g=1}^{G}\gamma_{ig}^{\left(m\right)}\log\;f_{g}\left(v_{i}|\varpi ,\alpha \right)$$

using backpropagation and gradient descent to obtain ${\hat{\theta }}_{g}^{\left(m\right)}=(\hat{\varpi },\hat{\alpha })$ , for g=1,2,…,G .

Let $\theta_{g,\text{long}}=\{\beta_{g},\tilde{\beta },\mu_{g},D_{g},\sigma^{2}\}$ be the longitudinal submodel parameters for class g . Then the density function of the longitudinal response $y_{i}$ conditional on class g is given by the following equation:

(37) $$f(y_{i}|D_{\text{long}},\theta_{g,\text{long}})=\phi_{n_{i}}(y_{i}|X_{i}\beta_{g}+{\tilde{X}}_{i}\tilde{\beta },Z_{i}\omega_{g}^{2}DZ_{i}^{⊺}+\sigma^{2}I_{n_{i}})$$

where $\phi_{n_{i}}$ denotes the density function of a $n_{i}$ -dimensional Gaussian density with mean vector $\mu =X_{i}\beta_{g}+{\tilde{X}}_{i}\tilde{\beta }$ , where $X_{i}$ is the design matrix for class-specific fixed effects with parameters $\beta_{g}=(\beta_{1g},\beta_{2g},\ldots ,\beta_{pg}{)}^{⊺}$ and ${\tilde{X}}_{i}$ is the design matrix for common fixed effects with parameters $\tilde{\beta }=({\tilde{\beta }}_{1},{\tilde{\beta }}_{2},\ldots ,{\tilde{\beta }}_{p}{)}^{⊺}$ . Further, the variance-covariance matrix is given by $V_{i}=Z_{i}\omega_{g}^{2}DZ_{i}^{⊺}+\sigma^{2}I_{n_{i}}$ , where $Z_{i}$ is the $n_{i}\times q$ design matrix for random effects, $\omega_{g}^{2}D$ is the class-specific variance-covariance matrix of the random effects, with $\omega_{G}^{2}=1$ for identifiability and $\sigma^{2}I_{n_{i}}$ is the residual variance-covariance matrix.

For the survival submodel, let $\theta_{g,\text{surv}}=\{\zeta_{g},\vartheta_{g},\vartheta \}$ be the survival submodel parameters for class g . We consider the Weibull proportional hazards model so that the class-specific hazard function is (adapted from equation (7)):

(38) $$h_{i}(t_{i}|D_{\text{surv}},\theta_{g,\text{surv}})=\zeta_{1g}\zeta_{2g}(t_{i}\zeta_{1g}{)}^{\zeta_{2g}-1}\exp\;\left(w_{i}^{⊺}\vartheta_{g}+{\tilde{w}}_{i}^{⊺}\vartheta \right)$$

where $\zeta_{1g}>0$ is the class-specific scale parameter, $\zeta_{2g}>0$ is the class-specific shape parameter, $w_{i}^{⊺}\vartheta_{g}$ represents class-specific covariate effects, ${\tilde{w}}_{i}^{⊺}\vartheta$ represents common covariate effects across all classes. The class-specific survival function is then given by the following equation:

(39) $$S_{i}(t_{i}|D_{\text{surv}},\theta_{g,\text{surv}})=\exp\;\left(-{\left(t_{i}\zeta_{1g}\right)}^{\zeta_{2g}}\exp\;\left(w_{i}^{⊺}\vartheta_{g}+{\tilde{w}}_{i}^{⊺}\vartheta \right)\right)$$

**Figure 2.**
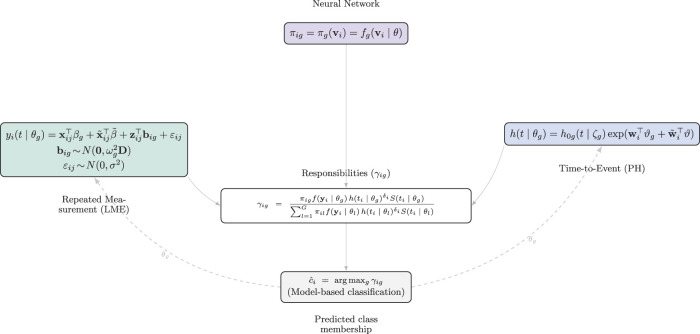
Neural network joint latent class model (JLCM) framework where the gating network is captured by a neural network as opposed to a multinomial model in the classical JLCM implementation.

The model parameters at each iteration (m) for both the survival and longitudinal submodels are estimated using the Marquardt–Levenberg algorithm (MLA).^39^ The MLA is applicable to optimization problems where a function F(θ) must be minimized (or equivalently, where L(θ)=−F(θ) is maximized) with respect to a set of m unconstrained parameters θ , provided that the second derivatives of F(θ) exist. In statistical contexts, the objective function is typically the deviance (to be minimized) or the log-likelihood (to be maximized). The improved MLA iteratively updates the parameter vector $\theta^{\left(k\right)}$ , starting from an initial value $\theta^{\left(0\right)}$ , until convergence according to the following equation:

(40) $$\theta^{\left(k+1\right)}=\theta^{\left(k\right)}-\psi_{1}^{\left(k\right)}{\left[\tilde{H}(F(\theta^{\left(k\right)}))\right]}^{-1}\nabla (F(\theta^{\left(k\right)}))$$

where $\theta^{\left(k\right)}$ denotes the parameter estimates at iteration k , $\nabla (F(\theta^{\left(k\right)}))$ is the gradient of the objective function, and $\tilde{H}(F(\theta^{\left(k\right)}))$ is a modified version of the Hessian matrix $H(F(\theta^{\left(k\right)}))$ , in which the diagonal elements are replaced by the following equation:

(41) $$\tilde{H}(F(\theta^{\left(k\right)}){)}_{ii}=H(F(\theta^{\left(k\right)}){)}_{ii}+\psi_{2}^{\left(k\right)}[(1-\psi_{3}^{\left(k\right)})|H(F(\theta^{\left(k\right)}){)}_{ii}|+\psi_{3}^{\left(k\right)},\text{tr}(H(F(\theta^{\left(k\right)})))]$$

In the standard MLA, the Hessian is inflated by a scaled identity matrix. Following Philipps et al.,^39^ this implementation instead applies curvature-based diagonal inflation, making the improved MLA an intermediate between the steepest descent and Newton methods. The scalars $\psi_{1}^{\left(k\right)}$ , $\psi_{2}^{\left(k\right)}$ , and $\psi_{3}^{\left(k\right)}$ denote iteration-specific hyperparameters that control the optimization step length, the Hessian inflation, and the curvature weighting, respectively. These quantities are determined at each iteration k as follows:

-
$\psi_{1}^{\left(k\right)}$ is set to 1 unless the objective function fails to decrease, in which case a line search identifies the optimal local step size.
-
$\psi_{2}^{\left(k\right)}$ and $\psi_{3}^{\left(k\right)}$ are internally adapted to ensure that
  1.
$\tilde{H}(F(\theta^{\left(k\right)}))$ remains positive definite at every iteration, and
  2.
$\tilde{H}(F(\theta^{\left(k\right)}))\to H(F(\theta^{\left(k\right)}))$ as $\theta^{\left(k\right)}\to \hat{\theta }$ .

When the optimization problem admits a unique solution, convergence to the minimum is guaranteed regardless of the initial parameter values.

The parameters of the neural network model are estimated using gradient descent as explained above.^33,40^ To summarize the NN-JLCM approach, we give the full algorithm in Algorithm 1 and showcase the intuition of the modelling approach in Figure 2.

**Table:**
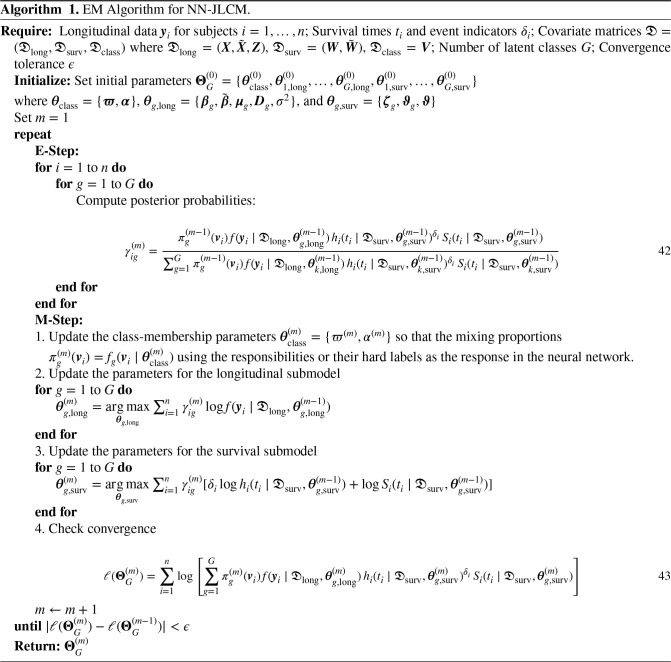


### 2.4. Optimal number of latent class assessment

We have assumed thus far that the number of latent classes is known, however, in practice, this is often unknown. In the latent class joint modelling literature, like in mixture modelling, there is unfortunately no one criteria for determining the number of classes, but a compromise among several criteria. Indeed, the resulting model with the ‘optimal’ number of classes tends to be the model which does not satisfy only one criteria, but a compromise among information criteria, classification meaningfulness and a valid conditional independence assumption as noted by Kyheng et al.^4^ and Proust-Lima et al.^9,10^ For a model to have a valid conditional independence assumption, the score test of conditional independence should not be significant.^32^

The information criteria, that is, the Bayesian information criterion (BIC)^41^ and the Akaike information criterion (AIC)^42^ are defined, respectively, as follows:

(44) BIC(G)=−2ℓ(θ)+log(N)×df(G),AIC(G)=−2ℓ(θ)+2×df(G)

where df(G) is the degrees of freedom and denotes the total number of parameters (as a function of the number of clusters G ), which consists of the G×p class-varying fixed-effects and the unique parameters of the Cholesky decomposition of the random effects matrix, comprising the longitudinal submodel, as well as the G×2 baseline hazard parameters of the survival submodel, along with the number of parameters of the weights and biases of the neural network, $p_{\mathrm{NN}}$ . Thus,

$$\mathrm{df}(G)=G\times p+G\times 2+p_{\mathrm{NN}}$$

The observed likelihood is denoted by ℓ(θ) , and N is the number of subjects under study. The AIC and BIC are among the most popular criteria to use for model selection of *G* in the mixture modelling literature.^43^

The integrated classification likelihood (ICL) of the model is computed as follows^9^:

(45) $$\mathrm{ICL}=\mathrm{BIC}-\sum_{i=1}^{N}\sum_{g=1}^{G}{\hat{\gamma }}_{ig}\log\;({\hat{\gamma }}_{ig})$$

which is a metric considering how discriminative the class predictions are. The ICL has been used to successfully determine the optimal number of clusters in finite mixture modelling approach.^44^

Further, we compute a measure of entropy^10^ alongside the ICL as another metric of classification ability. The metric is computed as follows:

(46) $$E=1+\frac{\sum_{i=1}^{N}\sum_{g=1}^{G}{\hat{\gamma }}_{ig}\log\;({\hat{\gamma }}_{ig})}{G\log\;(N)}$$

This metric is a value between 0 and 1 with scores closer to 1 indicating very good class discrimination ability.^9^

Finally, the responsibilities are used to evaluate the adequacy of the model through an *a posteriori* classification table. Each individual is assigned to the class corresponding to the largest responsibilities. For each class g=1,…,G , let $N_{g}$ denote its size. Then, for each set of observations that has been allocated to the gth class, compute the columnwise-mean of the responsibilities. As an example, for $c_{i}=1$ , and G=3 , there will be 3 means, as the resultant responsibilities are a $N_{G}\times G$ matrix. We then repeat append these results until we have the table in Table 1 with G columns and G rows. This Table 1 is presented, in general, as follows: Large values along the diagonal of this Table 1 indicate accurate classification and well-separated latent classes, whereas substantial off-diagonal values suggest potential class overlap or model misspecification. Classification of a model is considered satisfactory when all terms on the diagonal are above 80%.^4,9^ We report the average of the diagonal values (shortened to Avg. Diag.) as an additional measure alongside the ICL and entropy metrics.

## 3. Simulation study

In this section, we demonstrate the performance of the proposed estimation procedure for fitting the proposed NN-JLCM (combination of neural network and EM algorithm) compared to the traditional JLCM (combination of logistic model and Marquardt algorithm) approach. We consider B=1000 simulations for three sample sizes: n=100 , n=500 , and n=1000 . For each sample size, we simulate censored survival times conforming to light censoring ( r=0.05 ), moderate censoring ( r=0.25 ), and heavy censoring ( r=0.5 ), respectively. This results in nine combinations in total.

**Table 1.** Mean posterior class-membership probabilities by final class assignment.

		Mean of the probabilities of belonging to each class				
${\hat{c}}_{i}$	Size	1	⋯	g	⋯	G
1	$N_{1}$	$\frac{1}{N_{1}}\sum_{i=1}^{N_{1}}{\hat{\gamma }}_{i1}$	⋯	$\frac{1}{N_{1}}\sum_{i=1}^{N_{1}}{\hat{\gamma }}_{ig}$	⋯	$\frac{1}{N_{1}}\sum_{i=1}^{N_{1}}{\hat{\gamma }}_{iG}$
⋮		⋮		⋮		⋮
g	$N_{g}$	$\frac{1}{N_{g}}\sum_{i=1}^{N_{g}}{\hat{\gamma }}_{i1}$	⋯	$\frac{1}{N_{g}}\sum_{i=1}^{N_{g}}{\hat{\gamma }}_{ig}$	⋯	$\frac{1}{N_{g}}\sum_{i=1}^{N_{g}}{\hat{\gamma }}_{iG}$
⋮		⋮			⋱	⋮
G	$N_{G}$	$\frac{1}{N_{G}}\sum_{i=1}^{N_{G}}{\hat{\gamma }}_{i1}$	⋯	$\frac{1}{N_{G}}\sum_{i=1}^{N_{G}}{\hat{\gamma }}_{ig}$	⋯	$\frac{1}{N_{G}}\sum_{i=1}^{N_{G}}{\hat{\gamma }}_{iG}$

To assess the finite sample behaviour of the estimates, we compute the absolute bias (AB) and the mean squared error (MSE). The AB of the jth estimate of θ is

(47) $$\mathrm{AB}=\left|\frac{1}{B}\sum_{b=1}^{B}{\hat{\theta }}_{bj}-\theta_{j}\right|$$

where we consider B simulated estimates. Additionally, the MSE of the j th estimate is as follows:

(48) $$\mathrm{MSE}=\frac{1}{B}\sum_{b=1}^{B}{\left({\hat{\theta }}_{bj}-\theta_{j}\right)}^{2}$$

which average squared deviances between the jth estimate and true parameter over the B estimates.

The proposed model’s classification capability is also assessed by computing the classification error (CE) as follows:

$$\mathrm{CE}=\frac{1}{n}\sum_{i=1}^{n}I\left({\hat{c}}_{i}\neq c_{i}\right)$$

where $c_{i}$ is the true component label and ${\hat{c}}_{i}$ is the estimated label based on the estimated classification probability ${\hat{\gamma }}_{ig}$

$$\hat{c_{i}}=\underset{g}{\mathrm{argmax}}({\hat{\gamma }}_{ig}),\;i=1,\ldots ,n,g=1,\ldots ,G$$

The CE will be a value between 0 and 1, where 0 indicates that the assessed model shows a perfect classification ability and 1 indicates the perfect misclassification ability. Longitudinal predictions are evaluated using the MSE defined as follows:

(49) $${\mathrm{MSE}}_{y}=\frac{1}{\sum_{i=1}^{n}n_{i}}\sum_{i=1}^{n}\sum_{j=1}^{n_{i}}{\left({\hat{y}}_{ij}-y_{ij}\right)}^{2}$$

The MSE of the longitudinal recordings is evaluated on in-sample and out-of-sample data.

For time-to-event predictions, we use the integrated squared error (ISE) to measure the divergence between the true and the predicted survival curves. The ISE for a sample of n subjects is defined as follows:

$$\mathrm{ISE}=\frac{1}{n}\sum_{i=1}^{n}\frac{1}{\max_{i}T_{i}}\int_{0}^{\max_{j}T_{j}}{\left({\hat{S}}_{i}(t)-S_{i}(t)\right)}^{2}dt$$

where ${\hat{S}}_{i}(t)$ and $S_{i}(t)$ are the predicted and the true survival probability for subject i at time t , respectively. We compute ISE on in-sample subjects (ISE ${}_{\text{in}}$ ) and on out-of-sample subjects (ISE ${}_{\text{out}}$ ). In order to predict survival probabilities beyond subject i ’s event time $T_{i}$ , we assume that covariates for subject i remain unchanged since the last observed time.

### 3.1. Scenario I: Two-component model

#### 3.1.1. Data-generating process (DGP)

The settings of the first scenario are based on Kyheng et al.,^8^ where the authors considered a scenario inspired by a real dataset from Stamenic et al.,^45^ who used a JLCM as a prognostic tool for the prediction of graft failure in patients who are at risk of kidney graft loss. Stamenic et al.^45^ considered the serum creatine (SCr) as the longitudinal measurements of interest, and the time-to-event of interest was the onset of *de novo* donor-specific anti-HLA antibodies. Early prediction of graft failure in kidney transplants is crucial to assess the long-term success of kidney transplants.

We assume that the true survival times $T^{*}$ are governed by a two-component mixture Weibull distribution with scale parameter $\lambda_{g}$ and shape parameter $\kappa_{g}$ for g=1,2 . The survival probability is then computed as follows:

(50) $$S(t){|}_{\left(c_{i}=g\right)}=\exp\;\left(-{\left(\frac{t}{\lambda_{g}}\right)}^{\kappa_{g}}\right)$$

We assume that the right-censored times C follow a Weibull distribution with parameters $\tilde{\lambda }$ and $\tilde{\kappa }$ . We consider differing values for the scale and shape depending on the rate of censoring required, which we denote by r . This is given in Table 2.

**Table 2.** Varying parameters of the censoring distribution depending on censoring rate.

r	0.05	0.25	0.5
$\tilde{\kappa }$	10	10	0.5
$\tilde{\lambda }$	150	50	25

To simulate the longitudinal profiles of the SCr trajectories, we consider the following class-specific linear mixed-effects model with a random intercept

(51) $$\begin{array}{r} y_{ij}{|}_{c_{i}=g}=\beta_{0g}+\beta_{1g}t_{ij}+b_{ig}+\varepsilon_{ij}\\ \;b_{ig}∼N\left(0,\sigma_{b}^{2}\right),\;\varepsilon_{ij}∼N\left(0,\sigma_{\varepsilon }^{2}\right) \end{array}$$

We can group the fixed effects into vectors $\beta_{g}=(\beta_{1g},\beta_{2g}{)}^{⊺}$ . The full fixed-effects matrix over all clusters, $\beta =(\beta_{1},\beta_{2}{)}^{⊺}$ , is then

(52) $$\begin{array}{r} \beta =\left(\begin{array}{cc} 170 & 88\\ 100 & 1.2 \end{array}\right) \end{array}$$

We assume that the individual number of recordings ( $n_{i}$ ) follows a Poisson distribution with the average number of recordings being 5. The recording times are spread across $t_{ij}=1,3,6,12,18,24$ months. The class-specific random intercepts follow a zero-mean normal distribution with common variance over classes $\sigma_{b}^{2}$ , and we assume that $\sigma_{b}^{2}=50$ . The distribution of the marginal random effect for the ith subject is $b_{i}∼\sum_{g=1}^{G}\pi_{g}(x_{i})N(0,\sigma_{b}^{2}).$ Finally, the true error variance parameter is given as $\sigma_{\varepsilon }^{2}=60$ and does not vary by cluster.

To consider the effect of a mixing proportion which does not conform to assumptions of a monotonic linear function of a logistic regression, we consider the work of Huang and Yao,^21^ where the mixing proportions are governed by a sinusoidal function

(53) $$\pi_{1}(x_{i})=0.1+0.8\sin\;(\pi x_{i})\;\text{and}\;\pi_{2}(x_{i})=1-\pi_{1}(x_{i})$$

where $x_{i}$ is generated from a uniform distribution on the interval (0,1) .

#### 3.1.2. Results

**Figure 3.**
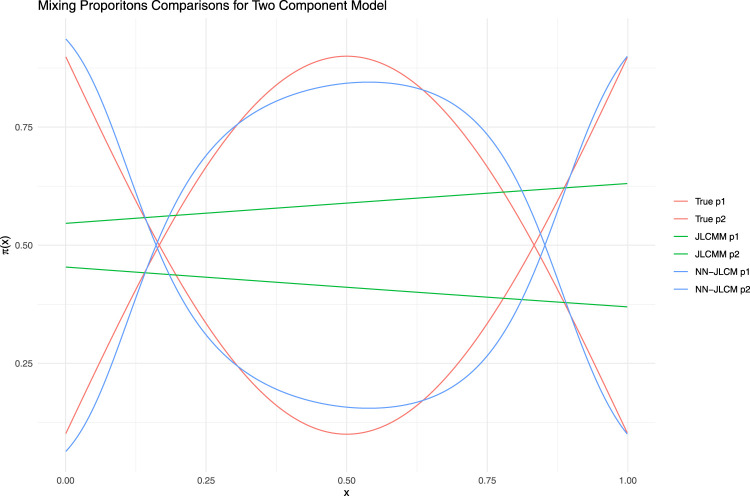
Mixing proportion comparisons for two-component model.

We have included the table of estimates under light censoring in Table 3 for 1000 simulations. The results for moderate and heavy censoring are included in Supplemental Tables S1 and S2, respectively. The performance for the model estimates of the submodels is similar across both methods, with similar estimate values, biases and MSEs. By increasing the sample size, the MSEs decrease; however, by increasing the censoring rate, the MSEs increase. Additionally, we plot the estimated mixing proportions for the NN-JLCM and JLCMM overlaid on the true mixing proportions in Figure 3.

**Table 3.** Bias and MSE results for NN-JLCM G=2 and JLCMM parameter estimates across sample sizes ( B=1000 simulations, 5% censoring).

			NN-JLCM			JLCMM		
Sample size	Parameter	Truth	Estimate	AB	MSE	Estimate	AB	MSE
*n* = 100	$\pi_{1}(x)$	–	–	0.014	0.007	–	0.282	0.117
	$\pi_{2}(x)$	–	–	0.014	0.007	–	0.282	0.117
	$\sigma_{e}$	7.746	7.734	0.012	0.060	7.733	0.013	0.059
	$\beta_{11}$	170	169.974	0.026	1.745	169.982	0.018	1.739
	$\beta_{12}$	88	87.976	0.024	0.682	87.974	0.026	0.691
	$\beta_{21}$	100	99.977	0.023	1.092	99.979	0.021	1.098
	$\beta_{22}$	1.2	1.213	0.013	0.381	1.209	0.009	0.380
	$d_{11}$	7.071	6.985	0.086	0.387	6.979	0.092	0.385
	$\kappa_{1}$	2	2.087	0.087	0.084	2.087	0.087	0.085
	$\kappa_{2}$	1.01	1.026	0.016	0.013	1.024	0.014	0.012
	$\lambda_{1}$	4.5	4.493	0.007	0.155	4.491	0.009	0.156
	$\lambda_{2}$	50	50.410	0.410	45.104	50.545	0.545	44.464
*n* = 500	$\pi_{1}(x)$	–	–	0.014	0.002	–	0.286	0.110
	$\pi_{2}(x)$	–	–	0.014	0.002	–	0.286	0.110
	$\sigma_{e}$	7.746	7.738	0.008	0.013	7.740	0.006	0.013
	$\beta_{11}$	170	170.031	0.031	0.381	170.031	0.031	0.382
	$\beta_{12}$	88	87.992	0.008	0.133	87.992	0.008	0.133
	$\beta_{21}$	100	100.010	0.010	0.220	100.011	0.011	0.219
	$\beta_{22}$	1.2	1.209	0.009	0.076	1.208	0.008	0.076
	$d_{11}$	7.071	7.060	0.011	0.071	7.060	0.011	0.071
	$\kappa_{1}$	2	2.016	0.016	0.014	2.016	0.016	0.014
	$\kappa_{2}$	1.01	1.014	0.004	0.002	1.014	0.004	0.002
	$\lambda_{1}$	4.5	4.493	0.007	0.029	4.493	0.007	0.029
	$\lambda_{2}$	50	49.958	0.042	9.141	49.954	0.046	9.134
*n* = 1000	$\pi_{1}(x)$	–	–	0.013	0.001	–	0.286	0.109
	$\pi_{2}(x)$	–	–	0.013	0.001	–	0.286	0.109
	$\sigma_{e}$	7.746	7.745	0.001	0.006	7.745	0.001	0.006
	$\beta_{11}$	170	169.995	0.005	0.163	169.995	0.005	0.163
	$\beta_{12}$	88	88.006	0.006	0.065	88.006	0.006	0.065
	$\beta_{21}$	100	99.980	0.020	0.113	99.980	0.020	0.113
	$\beta_{22}$	1.2	1.207	0.007	0.035	1.207	0.007	0.035
	$d_{11}$	7.071	7.051	0.020	0.039	7.051	0.020	0.039
	$\kappa_{1}$	2	2.010	0.010	0.007	2.010	0.010	0.007
	$\kappa_{2}$	1.01	1.013	0.003	0.001	1.013	0.003	0.001
	$\lambda_{1}$	4.5	4.504	0.004	0.015	4.504	0.004	0.015
	$\lambda_{2}$	50	50.039	0.039	4.454	50.039	0.039	4.453

MSE: mean squared error; NN-JLCM: neural-network joint latent class model; JLCMM: joint latent class mixed model; AB: absolute bias.

From Figure 3, we see that the JLCMM approach cannot handle the sinusoidal function as it does not conform to the assumptions of a logistic regression. This leads to biases in the classifications of the model. If the function for the mixing proportions is incorrect, then the posterior classifications naturally suffer. The neural network classification membership function of the NN-JLCM, however, performs quite well and captures the sinusoidal shape of the true function in equation (51), naturally leading to superior classification performance. This is confirmed by the AB and MSE of mixing proportion being much smaller relative to JLCMM across all sample sizes and rates of censoring in Table 3 and Supplemental Tables S1 and S2.

Finally, we computed the average misclassification error and the average prediction errors of the survival curves and longitudinal profiles over all B=1000 samples. The results are reported for all sample sizes and rates of censoring in Table 4. The misclassification error of JLCMM is 100% across all sample sizes and rates of censoring, while NN-JLCM retains a significant competitive advantage, having an average score of 0% misclassification.

**Table 4.** Performance metrics comparison: NN-JLCM versus JLCMM ( B=1000 simulations).

				ISE		MSE Y	
*n*	Cens.	Method	CE	In	Out	In	Out
100	5%	NN-JLCM	0.000	0.0014	0.0014	51.896	52.439
		JLCMM	1.000	0.1574	0.0014	51.892	19541.702
	25%	NN-JLCM	0.000	0.0017	0.0017	51.896	52.439
		JLCMM	1.000	0.1738	0.0017	51.900	19538.477
	50%	NN-JLCM	0.000	0.0018	0.0019	51.896	52.438
		JLCMM	1.000	0.1447	0.0019	51.897	19538.180
500	5%	NN-JLCM	0.000	0.0003	0.0003	51.857	51.958
		JLCMM	1.000	0.1586	0.0003	51.858	19555.589
	25%	NN-JLCM	0.000	0.0004	0.0004	51.857	51.958
		JLCMM	1.000	0.1746	0.0004	51.857	19555.636
	50%	NN-JLCM	0.000	0.0004	0.0004	51.857	51.958
		JLCMM	1.000	0.1443	0.0004	51.857	19555.499
1000	5%	NN-JLCM	0.000	0.0001	0.0001	51.945	51.997
		JLCMM	1.000	0.1585	0.0001	51.945	19557.277
	25%	NN-JLCM	0.000	0.0002	0.0002	51.945	51.997
		JLCMM	1.000	0.1745	0.0002	51.945	19557.277
	50%	NN-JLCM	0.000	0.0002	0.0002	51.945	51.997
		JLCMM	1.000	0.1442	0.0002	51.946	19557.384

NN-JLCM: neural-network joint latent class model; JLCMM: joint latent class mixed model; CE: classification error; ISE: integrated squared error; MSE: mean squared error.

The ISEs and MSEs are reported for both in-sample and out-of-sample data. The out-sample data had the same rate of censoring and number of subjects as the in-sample data. We can see that the in-sample performance of the JLCMM for ISE is quite poor relative to NN-JLCM across the study. The out-of-sample performance, interestingly, seems to be roughly equivalent between the two methods. The overall performance seems to improve slightly as n increases for JLCMM, but worsens as r increases.

For the prediction error, the in-sample performance is roughly equivalent across the methods, whereas the out-of-sample prediction error for JLCMM exhibits serious numerical issues, being more than 100 times greater than NN-JLCM. Increasing the sample size improves the performance slightly for both methods, considering only the in-sample cases. However, the out-of-sample performance actually seems to worsen for JLCMM as we increase the sample size, while the MSE improves for NN-JLCM.

This particular simulation setting showed that the NN-JLCM method is superior to the JLCMM method under varying forms of censoring for the mixing proporitons leading to better predictive and classification performance for NN-JLCM. Next, we will consider the case of three latent classes.

### 3.2. Scenario II: Three-component model

#### 3.2.1. Data-generating process

For the second simulation scenario, we consider a three-component model with a more complex mixing proportion taken from Xue and Yao.^19^ The mixing proportions are as follows:

(54) $$\begin{array}{rl} & \pi_{1}(x)=\frac{2\exp\;\left(-0.1x^{4}\right)}{1+\exp\;\left(-0.1x^{4}\right)}I(x<3)\\ & \pi_{2}(x)=\frac{2\exp\;\left(-0.1(x-6{)}^{4}\right)}{1+\exp\;\left(-0.1(x-6{)}^{4}\right)}I(x\geq 3)\\ & \pi_{3}(x)=1-\sum_{g=1}^{2}\pi_{g}(x) \end{array}$$

where each $x_{i}$ is generated from a uniform distribution on the interval (−5,12) . The longitudinal submodel is

(55) $$\begin{array}{rl} & y_{ij}{|}_{c_{i}=g}=x^{⊺}\beta_{g}+z^{⊺}b_{ig}+\varepsilon_{ij}\\ & b_{ig}∼N_{q}\left(0,\omega_{g}^{2}D\right),\;\varepsilon_{ij}∼N\left(0,\sigma_{\varepsilon }^{2}\right) \end{array}$$

where $\beta_{g}=(\beta_{1g},\beta_{2g}{)}^{⊺}$ , $b_{ig}=(b_{1ig},b_{2ig}{)}^{⊺}$ , and $x^{⊺}=z^{⊺}=(1,t_{ij}{)}^{⊺}$ . The recording times are made at strict intervals at 0, 6, 12, 18, and 24 months, respectively. The full fixed-effects matrix over all clusters, $\beta =(\beta_{1},\beta_{2},\beta_{3}{)}^{⊺}$ , is

(56) $$\begin{array}{r} \beta =\left(\begin{array}{cc} 1 & 0.5\\ 3 & 2\\ 2 & 1 \end{array}\right) \end{array}$$

The true variance parameter is given as $\sigma_{\varepsilon }^{2}=10$ and does not vary by cluster. The true values of the random effects variance-covariance matrix are given as follows:

(57) $$\begin{array}{r} D=\left(\begin{array}{cc} 9 & 3\\ 3 & 4 \end{array}\right) \end{array}$$

with the class-specifc proportions $\omega =(\omega_{1},\omega_{2})=(2.5,1.5)$ . We report the Cholesky decompositions of the variance-covariance matrix as that is what is directly estimated for both JLCMM and NN-JLCM. The Cholesky decomposition of equation (56) is

(58) $$\begin{array}{r} \tau =\left(\begin{array}{cc} 3 & 0\\ 1 & 1.732 \end{array}\right) \end{array}$$

Finally, we assume that the survival times are Weibull distributed with scale $\lambda_{g}$ and $\kappa_{g}$ with survival function given by equation (48). The full vector of scale parameters and shape parameters over all the clusters is given as λ=(4.5,50,25) and κ=(2,5,10). We assume that the right-censored times C follow a Weibull distribution with parameters $\tilde{\lambda }$ and $\tilde{\kappa }$ . Like before, we consider differing values for the scale and shape depending on the rate of censoring, given in Table 5.

**Table 5.** Varying parameters of the censoring distribution depending on censoring rate for three-component model.

r	0.05	0.25	0.5
$\tilde{\kappa }$	5	2	1.5
$\tilde{\lambda }$	65	50	30

#### 3.2.2. Results

In Table 6, we report the results of our 1000 samples generated using the DGP above under 5% censoring. The results for 25% and 50% censoring are reported in Supplemental Tables S3 and S4, respectively.

**Table 6.** Bias and MSE results for NN-JLCM G=3 and JLCMM parameter estimates across sample sizes ( B=1000 simulations, 5% censoring).

			NN-JLCM			JLCMM		
Sample size	Parameter	Truth	Estimate	AB	MSE	Estimate	AB	MSE
*n* = 100	$\pi_{1}(x)$	–	–	0.004	0.060	–	0.464	0.282
	$\pi_{2}(x)$	–	–	0.015	0.063	–	0.243	0.135
	$\pi_{3}(x)$	–	–	0.017	0.119	–	0.433	0.346
	$\sigma_{e}$	1.000	0.996	0.004	0.002	0.997	0.003	0.002
	$\beta_{11}$	0.500	0.483	0.017	2.853	0.486	0.014	2.866
	$\beta_{12}$	2.000	1.987	0.013	0.167	2.008	0.008	1.231
	$\beta_{21}$	3.000	3.001	0.001	1.061	2.973	0.027	1.108
	$\beta_{22}$	2.000	2.008	0.008	1.223	2.026	0.026	0.485
	$\beta_{31}$	2.000	2.050	0.050	0.470	1.982	0.018	0.183
	$\beta_{32}$	1.000	0.995	0.005	0.072	0.986	0.014	0.079
	$\tau_{11}$	3.000	2.960	0.040	0.078	2.944	0.056	0.090
	$\tau_{21}$	1.000	0.988	0.012	0.040	0.982	0.018	0.042
	$\tau_{22}$	1.732	1.704	0.028	0.023	1.694	0.039	0.028
	$\kappa_{1}$	2.000	2.154	0.154	0.208	2.155	0.155	0.216
	$\kappa_{2}$	5.000	5.705	0.705	2.635	5.603	0.603	3.750
	$\kappa_{3}$	10.000	10.248	0.248	1.257	10.361	0.361	1.722
	$\lambda_{1}$	4.500	4.466	0.034	0.266	4.471	0.029	0.297
	$\lambda_{2}$	50.000	50.205	0.205	5.970	49.548	0.452	12.388
	$\lambda_{3}$	25.000	24.985	0.015	0.106	25.005	0.005	0.132
	$\omega_{1}$	2.500	2.452	0.048	0.122	2.417	0.083	0.135
	$\omega_{2}$	1.500	1.490	0.010	0.045	1.634	0.134	0.116
*n* = 500	$\pi_{1}(x)$	–	–	0.003	0.017	–	0.495	0.279
	$\pi_{2}(x)$	–	–	0.006	0.018	–	0.271	0.129
	$\pi_{3}(x)$	–	–	0.009	0.035	–	0.469	0.340
	$\sigma_{e}$	1.000	1.000	0.000	0.000	1.000	0.000	0.000
	$\beta_{11}$	0.500	0.521	0.021	0.521	0.521	0.021	0.521
	$\beta_{12}$	2.000	2.006	0.006	0.035	1.988	0.012	0.237
	$\beta_{21}$	3.000	3.016	0.016	0.191	3.005	0.005	0.192
	$\beta_{22}$	2.000	1.988	0.012	0.237	1.999	0.001	0.088
	$\beta_{31}$	2.000	2.007	0.007	0.086	2.005	0.005	0.036
	$\beta_{32}$	1.000	1.007	0.007	0.015	1.004	0.004	0.015
	$\tau_{11}$	3.000	2.990	0.010	0.014	2.986	0.014	0.015
	$\tau_{21}$	1.000	0.995	0.005	0.008	0.994	0.006	0.008
	$\tau_{22}$	1.732	1.726	0.006	0.005	1.724	0.009	0.005
	$\kappa_{1}$	2.000	2.027	0.027	0.026	2.027	0.027	0.028
	$\kappa_{2}$	5.000	5.128	0.128	0.243	5.031	0.031	0.433
	$\kappa_{3}$	10.000	10.064	0.064	0.221	10.101	0.101	0.274
	$\lambda_{1}$	4.500	4.492	0.008	0.054	4.493	0.007	0.056
	$\lambda_{2}$	50.000	50.079	0.079	1.194	49.844	0.156	1.883
	$\lambda_{3}$	25.000	25.000	0.000	0.023	24.997	0.003	0.027
	$\omega_{1}$	2.500	2.492	0.008	0.023	2.486	0.014	0.023
	$\omega_{2}$	1.500	1.493	0.007	0.008	1.670	0.170	0.047
*n* = 1000	$\pi_{1}(x)$	–	–	0.003	0.009	–	0.500	0.278
	$\pi_{2}(x)$	–	–	0.005	0.010	–	0.276	0.129
	$\pi_{3}(x)$	–	–	0.008	0.019	–	0.475	0.339
	$\sigma_{e}$	1.000	1.000	0.000	0.000	1.000	0.000	0.000
	$\beta_{11}$	0.500	0.501	0.001	0.239	0.501	0.001	0.239
	$\beta_{12}$	2.000	1.997	0.003	0.017	1.997	0.003	0.119
	$\beta_{21}$	3.000	2.990	0.010	0.104	2.985	0.015	0.109
	$\beta_{22}$	2.000	1.998	0.002	0.119	1.995	0.005	0.043
	$\beta_{31}$	2.000	2.001	0.001	0.042	1.996	0.004	0.018
	$\beta_{32}$	1.000	0.996	0.004	0.007	0.994	0.006	0.007
	$\tau_{11}$	3.000	2.996	0.004	0.007	2.993	0.007	0.008
	$\tau_{21}$	1.000	0.998	0.002	0.004	0.998	0.002	0.004
	$\tau_{22}$	1.732	1.727	0.005	0.002	1.726	0.006	0.003
	$\kappa_{1}$	2.000	2.013	0.013	0.013	2.012	0.012	0.013
	$\kappa_{2}$	5.000	5.095	0.095	0.117	5.007	0.007	0.210
	$\kappa_{3}$	10.000	10.021	0.021	0.115	10.050	0.050	0.142
	$\lambda_{1}$	4.500	4.502	0.002	0.026	4.504	0.004	0.027
	$\lambda_{2}$	50.000	50.038	0.038	0.551	49.860	0.140	0.904
	$\lambda_{3}$	25.000	24.999	0.001	0.011	24.996	0.004	0.012
	$\omega_{1}$	2.500	2.494	0.006	0.010	2.492	0.008	0.011
	$\omega_{2}$	1.500	1.500	0.000	0.004	1.664	0.164	0.036

MSE: mean squared error; NN-JLCM: neural-network joint latent class model; JLCMM: joint latent class mixed model; AB: absolute bias.

The performance of the estimates of the longitudinal model for NN-JLCM seems to be roughly equivalent to JLCMM, showcasing equivalent MSEs as n increases. As r increases, the MSEs slightly increase for both NN-JLCM and JLCMM. The MSEs for the survival model for NN-JLCM are much lower than those for JLCM, even as n increases. The difference is exacerbated further as r increases, with JLCMM experiencing difficulties in estimating the survival parameters reliably at smaller sample sizes. The MSEs and biases increase as well for NN-JLCM, however, not to the same degree as JLCMM. Additionally, from the results in the tables, it is clear that the AB and MSE for the mixing proportions for NN-JLCM are superior to those of JLCMM. As n increases, the performance for JLCMM deteriorates further with higher biases. The performance for the mixing proportions for this scenario seems to be largely unaffected by the degree of censoring.

**Figure 4.**
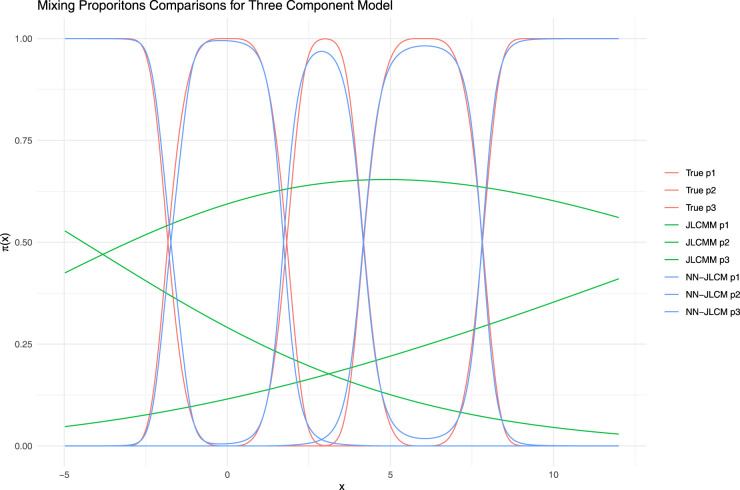
Mixing proportion comparisons.

In Figure 4, we plotted the fitted mixing proportions ${\hat{\pi }}_{g}(x)$ for NN-JLCM and JLCMM over the true mixing proportions $\pi_{g}(x)$ . From Figure 4, it is clear that the neural network class-membership model of NN-JLCM yields a superior fit in comparison to JLCMM, with the latter unable to capture the highly non-monotonic and nonlinear nature of the true mixing proportions.

Finally, in Table 7, we report the prediction errors for the survival curves and longitudinal profiles and the CEs. CEs for JLCMM and NN-JLCM are similar to before, with NN-JLCM greatly outperforming JLCMM. As the level of censoring increases (i.e. r increases) the misclassification increases very slightly for NN-JLCM but it is significantly worse for JLCM. The ISEs and MSEs are similar to Scenario I as well, with NN-JLCM showing better predictive performance. However, the ISE for the out-of-sample predictions for JLCMM is much worse than NN-JLCM. The prediction MSEs are equivalent for in-sample predictions, but the JLCMM performs poorly on out-of-sample predictions. The prediction MSEs seem to be fairly robust to censoring, with minimal change as r increases for both models.

**Table 7.** Performance metrics comparison G=3 : NN-JLCM versus JLCMM ( B=1000 simulations).

				ISE		MSE Y	
*n*	Cens.	Method	CE	In	Out	In	Out
100	5%	NN-JLCM	0.010	0.0018	0.0043	0.576	0.579
		JLCMM	0.806	0.2200	0.0107	0.577	2178.314
	25%	NN-JLCM	0.020	0.0024	0.0067	0.576	0.578
		JLCMM	0.831	0.2654	0.0186	0.576	2183.334
	50%	NN-JLCM	0.038	0.0030	0.0096	0.576	0.578
		JLCMM	0.852	0.2960	0.0402	0.576	2180.963
500	5%	NN-JLCM	0.008	0.0004	0.0011	0.579	0.580
		JLCMM	0.805	0.2145	0.0082	0.579	2115.339
	25%	NN-JLCM	0.015	0.0005	0.0017	0.579	0.580
		JLCMM	0.832	0.2663	0.0151	0.579	2115.842
	50%	NN-JLCM	0.027	0.0006	0.0022	0.579	0.580
		JLCMM	0.866	0.3144	0.0223	0.579	2120.313
1000	5%	NN-JLCM	0.008	0.0003	0.0006	0.579	0.579
		JLCMM	0.805	0.2137	0.0079	0.578	2118.937
	25%	NN-JLCM	0.015	0.0003	0.0009	0.579	0.579
		JLCMM	0.832	0.2663	0.0148	0.578	2110.841
	50%	NN-JLCM	0.026	0.0003	0.0012	0.578	0.579
		JLCMM	0.866	0.3165	0.0215	0.577	2109.102

NN-JLCM: neural-network joint latent class model; JLCMM: joint latent class mixed model; CE: classification error; ISE: integrated squared error; MSE: mean squared error.

In this scenario, we see that NN-JLCM once again outperforms JLCMM in predictive accuracy and parameter stability across censoring rates and sample sizes. In the next scenario, we consider a four-component model.

### 3.3. Scenario III: Four-component model

#### 3.3.1. Data-generating process

For the third Scenario, we consider a four-component model with complex mixing proportions extending the framework from Xue and Yao.^19^ The mixing proportions are as follows:

(59) $$\begin{array}{rl} & \pi_{1}(x)=\frac{2\exp\;\left(-0.1x^{4}\right)}{1+\exp\;\left(-0.1x^{4}\right)}I(x<3)\\ & \pi_{2}(x)=1-\frac{2\exp\;\left(-0.1x^{4}\right)}{1+\exp\;\left(-0.1x^{4}\right)}I(x<0)\\ & \pi_{3}(x)=\frac{2\exp\;\left(-0.1(x-6{)}^{4}\right)}{1+\exp\;\left(-0.1(x-6{)}^{4}\right)}I(x\geq 3)\\ & \pi_{4}(x)=1-\sum_{g=1}^{3}\pi_{g}(x) \end{array}$$

where each $x_{i}$ is generated from a uniform distribution on the interval (−5,12) .

The true parameters of the survival and longitudinal submodels are inspired by the PAQUID dataset. The PAQUID dataset consists of a random subsample of 500 subjects from the study of a French cohort about dementia and Alzheimer’s incidence rates.^27^ The objective here is to jointly model the repeated measurements of scores for the MMSE, which is an assessment of cognitive performance, and the event times for dementia occurrence. We simulate synthetic data for MMSE using the following longitudinal submodel structure:

(60) $$\begin{array}{rl} & y_{ij}{|}_{c_{i}=g}=x_{ij}^{⊺}\beta_{g}+z_{ij}^{⊺}b_{ig}+\varepsilon_{ij}\\ & b_{ig}∼N_{q}\left(0,D\right),\;\varepsilon_{ij}∼N\left(0,\sigma_{\varepsilon }^{2}\right) \end{array}$$

where $\beta_{g}=(\beta_{1g},\beta_{2g},\beta_{3g}{)}^{⊺}$ , $b_{ig}=(b_{1ig},b_{2ig},b_{3ig}{)}^{⊺}$ , and $x_{ij}^{⊺}=z_{ij}^{⊺}=(1,t_{ij},t_{ij}^{2}{)}^{⊺}$ . The full fixed-effects matrix over all clusters, $\beta =(\beta_{1},\beta_{2},\beta_{3},\beta_{4}{)}^{⊺}$ , is

(61) $$\begin{array}{r} \beta =\left(\begin{array}{ccc} 66 & 58 & 65\\ 83 & 14 & 19\\ 5 & -66 & -16\\ -12 & -3 & 17 \end{array}\right) \end{array}$$

The true variance parameter is given as $\sigma_{\varepsilon }^{2}=10$ and does not vary by cluster. The true values of the random effects variance-covariance matrix are given as follows:

(62) $$\begin{array}{r} D=\left(\begin{array}{ccc} 236 & -253 & 76\\ -253 & 442 & -141\\ 76 & -141 & 46 \end{array}\right) \end{array}$$

We report the Cholesky decompositions of the variance-covariance matrix, as that is what is directly estimated for both JLCMM and NN-JLCM. The Cholesky decomposition of equation (60) is

(63) $$\begin{array}{r} \tau =\left(\begin{array}{ccc} 15.362 & 0 & 0\\ -16.469 & 13.068 & 0\\ 4.947 & -4.555 & 0.882 \end{array}\right) \end{array}$$

Finally, we assume that the survival times are Weibull distributed with scale $\lambda_{g}$ and shape $\kappa_{g}$ with survivor function given by equation (48). The full vector of scale parameters and shape parameters over all the clusters is given as λ=(76,98,89,83) and κ=(30,40,43,39).

We assume that the right-censored times C follow a Weibull distribution with parameters $\tilde{\lambda }$ and $\tilde{\kappa }$ . Like before, we consider differing values for the scale and shape depending on the rate of censoring, given in Table 8.

**Table 8.** Varying parameters of the censoring distribution depending on censoring rate for four-component model.

r	0.05	0.25	0.5
$\tilde{\kappa }$	5	3	2
$\tilde{\lambda }$	150	125	100

**Table 9.** Bias and MSE results for NN-JLCM G=4 and JLCMM parameter estimates across sample sizes ( B=1000 simulations, 5% censoring) – part 1.

			NN-JLCM			JLCMM		
Sample size	Parameter	Truth	Estimate	AB	MSE	Estimate	AB	MSE
*n* = 100	$\pi_{1}(x)$	–	–	0.005	0.061	–	0.324	0.335
	$\pi_{2}(x)$	–	–	0.003	0.026	–	0.336	0.330
	$\pi_{3}(x)$	–	–	0.011	0.064	–	0.291	0.314
	$\pi_{4}(x)$	–	–	0.012	0.094	–	0.384	0.392
	$\sigma_{e}$	10	9.936	0.064	0.187	9.949	0.051	0.197
	$\beta_{11}$	66	66.233	0.233	16.834	68.878	2.878	88.963
	$\beta_{12}$	58	57.995	0.005	19.882	−10.367	68.367	5561.855
	$\beta_{13}$	65	64.977	0.023	15.819	−2.563	67.563	4702.963
	$\beta_{21}$	83	83.081	0.081	8.663	68.260	14.740	302.553
	$\beta_{22}$	14	13.708	0.292	44.260	−8.862	22.862	1378.190
	$\beta_{23}$	19	19.197	0.197	49.652	−3.128	22.128	617.682
	$\beta_{31}$	5	5.120	0.120	41.464	67.945	62.945	4033.319
	$\beta_{32}$	−66	−66.011	0.011	22.615	−5.919	60.081	4254.615
	$\beta_{33}$	−16	−15.921	0.079	5.888	−4.726	11.274	247.771
	$\beta_{41}$	−12	−12.041	0.041	6.587	68.260	80.260	6527.045
	$\beta_{42}$	−3	−3.019	0.019	5.556	−8.862	5.862	889.890
	$\beta_{43}$	17	17.003	0.003	3.051	−3.128	20.128	533.169
	$\tau_{11}$	15.362	14.816	0.547	2.939	16.050	0.687	3.636
	$\tau_{21}$	−16.469	−15.614	0.855	11.127	−23.610	7.141	80.243
	$\tau_{31}$	4.947	4.641	0.306	1.500	7.064	2.117	7.916
	$\tau_{22}$	13.068	12.196	0.872	4.413	20.108	7.039	66.298
	$\tau_{32}$	−4.555	−4.170	0.385	0.974	−6.480	1.925	6.732
	$\tau_{33}$	0.882	0.367	0.514	0.538	0.787	0.095	0.532
	$\kappa_{1}$	30	32.489	2.489	52.342	25.249	4.751	143.398
	$\kappa_{2}$	40	44.119	4.119	121.064	26.441	13.559	277.629
	$\kappa_{3}$	43	47.092	4.092	109.847	26.658	16.342	346.877
	$\kappa_{4}$	39	40.582	1.582	31.879	26.779	12.221	233.559
	$\lambda_{1}$	76	75.927	0.073	0.341	†	†	†
	$\lambda_{2}$	98	97.971	0.029	0.412	$3.92\times 10^{84}$	$3.92\times 10^{84}$	$1.48\times 10^{169}$
	$\lambda_{3}$	89	88.980	0.020	0.263	157611.856	157522.856	$2.14\times 10^{13}$
	$\lambda_{4}$	83	82.981	0.019	0.122	$2.38\times 10^{182}$	$2.38\times 10^{182}$	†
*n* = 500	$\pi_{1}(x)$	–	–	0.003	0.017	–	0.350	0.338
	$\pi_{2}(x)$	–	–	0.002	0.007	–	0.345	0.328
	$\pi_{3}(x)$	–	–	0.004	0.017	–	0.328	0.321
	$\pi_{4}(x)$	–	–	0.005	0.027	–	0.387	0.370
	$\sigma_{e}$	10	10.000	0.000	0.035	10.002	0.002	0.037
	$\beta_{11}$	66	65.954	0.046	3.309	68.023	2.023	83.372
	$\beta_{12}$	58	58.024	0.024	3.626	−8.020	66.020	5287.799
	$\beta_{13}$	65	64.968	0.032	3.053	−3.569	68.569	4856.369
	$\beta_{21}$	83	83.013	0.013	1.828	67.624	15.376	317.078
	$\beta_{22}$	14	14.116	0.116	7.687	−6.840	20.840	1422.281
	$\beta_{23}$	19	18.948	0.052	9.037	−3.967	22.967	678.117
	$\beta_{31}$	5	5.015	0.015	7.880	71.736	66.736	4534.420
	$\beta_{32}$	−66	−66.010	0.010	4.277	−17.541	48.459	3073.557
	$\beta_{33}$	−16	−16.044	0.044	0.996	−0.086	15.914	416.223
	$\beta_{41}$	−12	−11.982	0.018	1.192	67.624	79.624	6420.726
	$\beta_{42}$	−3	−2.999	0.001	1.025	−6.840	3.840	1002.716
	$\beta_{43}$	17	17.005	0.005	0.542	−3.967	20.967	590.251
	$\tau_{11}$	15.362	15.250	0.112	0.507	16.367	1.005	1.721
	$\tau_{21}$	−16.469	−16.310	0.159	1.983	−23.858	7.389	65.237
	$\tau_{31}$	4.947	4.894	0.053	0.265	7.148	2.200	5.848
	$\tau_{22}$	13.068	12.858	0.210	0.710	20.524	7.456	63.946
	$\tau_{32}$	−4.555	−4.462	0.093	0.157	−6.575	2.019	5.505
	$\tau_{33}$	0.882	0.789	0.093	0.055	1.052	0.170	0.098
	$\kappa_{1}$	30	30.433	0.433	6.182	25.790	4.210	209.812
	$\kappa_{2}$	40	40.552	0.552	12.485	30.692	9.308	137.082
	$\kappa_{3}$	43	43.734	0.734	12.744	30.763	12.237	201.069
	$\kappa_{4}$	39	39.342	0.342	5.602	31.569	7.431	106.027
	$\lambda_{1}$	76	75.984	0.016	0.069	†	†	†
	$\lambda_{2}$	98	97.991	0.009	0.080	91.007	6.993	10155.378
	$\lambda_{3}$	89	89.001	0.001	0.045	$1.28\times 10^{19}$	$1.28\times 10^{19}$	$7.96\times 10^{37}$
	$\lambda_{4}$	83	82.998	0.002	0.026	87.921	4.921	421.241

MSE: mean squared error; NN-JLCM: neural-network joint latent class model; JLCMM: joint latent class mixed model; AB: absolute bias.

† indicates infinite or extremely large values due to numerical instability.

**Table 10.** Bias and MSE results for NN-JLCM G=4 and JLCMM parameter estimates across sample sizes ( B=1000 simulations, 5% censoring) – part 2.

			NN-JLCM			JLCMM		
Sample size	Parameter	Truth	Estimate	AB	MSE	Estimate	AB	MSE
*n* = 1000	$\pi_{1}(x)$	–	–	0.003	0.009	–	0.361	0.348
	$\pi_{2}(x)$	–	–	0.001	0.004	–	0.351	0.335
	$\pi_{3}(x)$	–	–	0.004	0.010	–	0.340	0.330
	$\pi_{4}(x)$	–	–	0.005	0.015	–	0.369	0.347
	$\sigma_{e}$	10	10.003	0.003	0.018	10.002	0.002	0.019
	$\beta_{11}$	66	66.023	0.023	1.595	67.231	1.231	70.399
	$\beta_{12}$	58	58.007	0.007	1.841	−4.964	62.964	4801.205
	$\beta_{13}$	65	65.015	0.015	1.642	−4.788	69.788	5011.354
	$\beta_{21}$	83	83.009	0.009	0.905	67.210	15.790	317.449
	$\beta_{22}$	14	13.968	0.032	3.979	−4.726	18.726	1174.304
	$\beta_{23}$	19	19.001	0.001	4.511	−4.895	23.895	705.896
	$\beta_{31}$	5	4.973	0.027	4.063	72.701	67.701	4675.610
	$\beta_{32}$	−66	−66.007	0.007	2.179	−23.344	42.656	2585.157
	$\beta_{33}$	−16	−15.988	0.012	0.520	2.387	18.387	512.912
	$\beta_{41}$	−12	−12.009	0.009	0.576	67.210	79.210	6342.304
	$\beta_{42}$	−3	−2.994	0.006	0.504	−4.726	1.726	826.621
	$\beta_{43}$	17	17.006	0.006	0.286	−4.895	21.895	614.315
	$\tau_{11}$	15.362	15.300	0.063	0.246	16.513	1.151	1.711
	$\tau_{21}$	−16.469	−16.358	0.111	0.976	−24.436	7.967	69.898
	$\tau_{22}$	4.947	4.912	0.035	0.130	7.236	2.288	5.810
	$\tau_{31}$	13.068	12.964	0.104	0.342	21.046	7.978	68.555
	$\tau_{32}$	−4.555	−4.507	0.048	0.074	−6.546	1.991	4.662
	$\tau_{33}$	0.882	0.843	0.038	0.018	1.118	0.236	0.080
	$\kappa_{1}$	30	30.233	0.233	2.967	25.143	4.857	215.849
	$\kappa_{2}$	40	40.398	0.398	5.981	30.222	9.778	145.261
	$\kappa_{3}$	43	43.411	0.411	6.004	30.053	12.947	214.138
	$\kappa_{4}$	39	39.127	0.127	2.751	30.317	8.683	118.937
	$\lambda_{1}$	76	75.997	0.003	0.034	$8.78\times 10^{195}$	$8.78\times 10^{195}$	†
	$\lambda_{2}$	98	97.998	0.002	0.036	107.615	9.615	406670.734
	$\lambda_{3}$	89	89.005	0.005	0.023	13284.814	13195.814	$1.66\times 10^{11}$
	$\lambda_{4}$	83	82.995	0.005	0.013	88.018	5.018	746.403

MSE: mean squared error; NN-JLCM: neural-network joint latent class model; JLCMM: joint latent class mixed model; AB: absolute bias.

† indicates infinite or extremely large values due to numerical instability.

#### 3.3.2. Results

The results for 5% censoring for n=100,500,1000 are reported in Tables 9 and 10. The results for 25% and 50% censoring are reported in Supplemental Tables S5 to S8.

The performance of the estimates of the longitudinal model for NN-JLCM appears to be roughly equivalent to JLCMM, showcasing comparable MSEs as n increases. As r increases, the MSEs slightly increase for both NN-JLCM and JLCMM. However, the MSEs for the survival model parameters show a marked difference between the two approaches. The NN-JLCM demonstrates significantly lower MSEs than JLCMM, even as n increases. This difference becomes more pronounced as r increases, with JLCMM experiencing difficulties in estimating the survival parameters reliably at smaller sample sizes.

The survival parameter estimates for JLCMM exhibit severe numerical instability, particularly evident in the extreme values marked with † symbols indicating infinite or extremely large estimates. For instance, at n=100 with 5% censoring, JLCMM produces estimates such as $\lambda_{2}=3.92\times 10^{84}$ and $\lambda_{4}=2.38\times 10^{182}$ , demonstrating complete breakdown of the estimation procedure. These numerical instabilities persist across different sample sizes and censoring rates, though they become somewhat less frequent as n increases.

In contrast, NN-JLCM maintains accurate parameter estimates throughout all sample sizes and censoring rates in this scenario. The MSEs and biases for NN-JLCM show the expected decrease as sample size increases. This stability is particularly noteworthy given the complexity of the four-component model with its highly non-monotonic mixing proportions.

**Figure 5.**
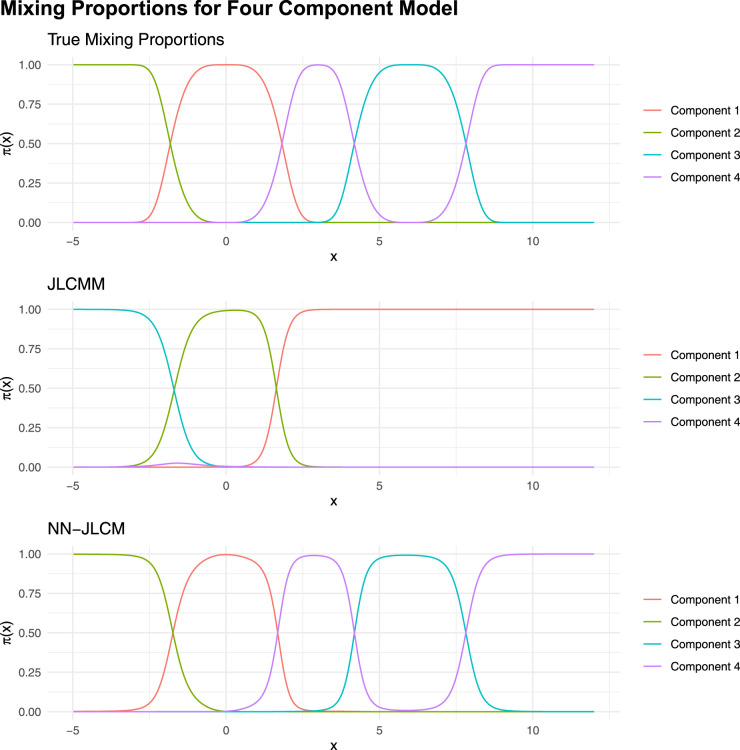
Mixing proportion comparisons for four-component model.

The mixing proportion estimates reveal the core advantage of making use of a neural network-based class-membership model. The NN-JLCM achieves lower bias and MSE values compared to the JLCMM, which barely improves with increased sample size. By looking at Figure 5, this showcases the poor performance of the logistic regression class-membership model of the JLCMM to capture the complex pattern of the true mixing proportions, which propagates through to all other parameter estimates in the JLCMM model.

Finally, examining Table 11, the performance metrics demonstrate the practical consequences of these estimation differences. The NN-JLCM achieves near-perfect classification with misclassification errors ranging from 0.005 to 0.017 across all scenarios, while JLCMM exhibits poor classification performance with errors between 0.756 and 0.794. The ISE and MSE metrics follow similar patterns, with NN-JLCM showing superior predictive accuracy for both survival curves and longitudinal trajectories. The out-of-sample prediction errors for JLCMM are large, often exceeding 500 times those of NN-JLCM, indicating potential overfitting and poor generalization capability.

**Table 11.** Performance metrics comparison: NN-JLCM versus JLCMM ( B=1000 simulations).

				ISE		MSE Y	
*n*	Cens.	Method	CE	In	Out	In	Out
100	5%	NN-JLCM	0.007	0.0018	0.0041	72.055	77.366
		JLCMM	0.794	0.3170	0.2029	67.374	674.309
	25%	NN-JLCM	0.011	0.0022	0.0063	71.921	77.264
		JLCMM	0.793	0.2612	0.1792	67.696	607.540
	50%	NN-JLCM	0.017	0.0025	0.0074	71.809	77.351
		JLCMM	0.788	0.1928	0.1448	67.514	620.108
500	5%	NN-JLCM	0.005	0.0004	0.0008	71.582	72.440
		JLCMM	0.780	0.3045	0.1688	67.062	567.264
	25%	NN-JLCM	0.008	0.0005	0.0013	71.450	72.332
		JLCMM	0.789	0.2587	0.1600	66.657	2615.259
	50%	NN-JLCM	0.013	0.0005	0.0017	71.266	72.190
		JLCMM	0.794	0.1897	0.1330	66.567	540.143
1000	5%	NN-JLCM	0.005	0.0002	0.0004	71.386	71.792
		JLCMM	0.775	0.3023	0.1654	66.560	565.671
	25%	NN-JLCM	0.008	0.0002	0.0007	71.251	71.671
		JLCMM	0.771	0.2570	0.1579	66.422	1335.263
	50%	NN-JLCM	0.013	0.0003	0.0008	71.094	71.529
		JLCMM	0.756	0.1829	0.1296	66.331	496.024

NN-JLCM: neural-network joint latent class model; JLCMM: joint latent class mixed model; CE: classification error; ISE: integrated squared error; MSE: mean squared error.

This four-component scenario represents the most challenging test case, and the results clearly demonstrate that NN-JLCM provides a robust and flexible alternative to traditional JLCMM when the mixing proportions deviate significantly from logistic regression assumptions.

## 4. Additional study of finite sample properties

In this section, we conduct a series of simulation experiments to evaluate the performance of the proposed NN-JLCM against the standard JLCM, the SREM implemented in the JM package, and the JLCT method. The experiments are organised as follows. First, we assess the sensitivity of each method to the number of latent classes by fitting models across a range of specified values of G and evaluating goodness-of-fit and classification accuracy. Second, we examine model behaviour under weak class separation, where the class-specific parameters are close in value, and cluster overlap is substantial. Third, we generate data from a known SREM process and fit the NN-JLCM, JLCM, and JLCT to evaluate performance under a true violation of the conditional independence assumption. Here, the score test serves as a diagnostic to assess whether each latent class method can detect the departure from conditional independence. Finally, we consider the converse scenario, generating data from the proposed NN-JLCM and fitting all competing methods. This provides a complementary assessment of the score test: under a true JLCM DGP, the test should fail to reject conditional independence, confirming that it is well-calibrated in both directions.

### 4.1. Survival and longitudinal submodel predictive assessments

To assess survival curves, we use the integrated Brier score (BS) for censored data. Let $\hat{G}(t)$ denote the Kaplan-Meier estimate of the censoring distribution, that is, the Kaplan-Meier estimate based on observations $\left(t_{i},1-\delta_{i}\right)$ , i=1,…,n (in the absence of ties between censored and uncensored observations). The BS as a function of time t>0 is defined by Yao et al.^46^ as follows:

(64) $$\mathrm{BS}\;(t)=\frac{1}{N}\sum_{i=1}^{N}\left(\frac{\hat{S}{\left(t|w_{i}\right)}^{2}I\left(t_{i}⩽t\right)\delta_{i}}{\hat{G}\left(t_{i}\right)}+\frac{{\left(1-\hat{S}\left(t|w_{i}\right)\right)}^{2}I\left(t_{i}>t\right)}{\hat{G}(t)}\right)$$

where $\hat{S}(t|w_{i})$ is the predicted survival curve at time t conditional on the ith subjects’ covariates $w_{i}$ , and $I(t_{i}>t)$ denotes the empirical survival function at time and the integrated BS (IBS) is therefore defined as^47^

(65) $$\mathrm{IBS}=\frac{1}{\max\left(t_{i}\right)}\int_{0}^{\max\left(t_{i}\right)}\mathrm{BS}(t)dt$$

The IBS yields a bounded value between 0 and 1. Scores closer to 0 imply a better fit of the proposed model to the empirical survival function and vice versa. Further, we compute the MSE of the prediction (see equation (47)) for both in-sample and out-of-sample datapoints.

### 4.2. Alternate techniques for joint modelling

In addition to the JLCMM and the proposed model, the NN-JLCM, we consider alternate joint modelling techniques for the application. The first among these we consider is the SREM, which is a popular approach for jointly modelling time-to-event processes and longitudinal processes when the direct association between the two is of interest. This is due to the hazard function incorporating an additional term for the mean of the longitudinal profile, allowing us to compute the effect on the risk of event-failure under a marginal increase in the subject-specific mean profile. We use the well-established JM package for estimation,^7,26^ which uses an EM algorithm to find MLEs. For further information about SREMs, we refer the reader to Rizopoulos^7^, Hickey et al.^6^ and Papageorgiou et al.^12^

Secondly, we consider the JLCT proposed by Zhang and Simonoff.^17^ The reason for this is that it is the only other joint latent class modelling approach which considers an embedded machine learning approach for the class-membership submodel. The approach fits a decision tree to the data to find latent class partitions according to the assumption of conditional independence within latent classes. The decision tree recursively does this using a log-likelihood ratio test to verify statistical independence using the extended Cox model which includes the longitudinal measurement of interest within each node. If the parameter on the longitudinal measurement is not significantly different from zero, then it means that there is statistical independence within obtained partition. The goal is to find segments of the data in which the children’s nodes are more homogeneous than their parent nodes to conform to statistical independence within the clusters. Once these partitions are obtained, they are used to fit a separate survival regression model with class-specific parameters and a linear mixed-effects model with class-specific random effects and independent subject-specific random effects. Additionally, JLCT allowed for time-varying latent classifications. The JLCT showcased superior predictive performance and modelling flexibility when compared to SREM and JLCM in a simulation study and an application to PAQUID.^17,48^ For further information about JLCT, we refer the reader to the work of Zhang and Simonoff^17,48^ and Fu and Simonoff.^49^

### 4.3. Multiple- G simulation

In this simulation, we consider a similar approach to Nguyen and McLachlan^50^ of showcasing the efficacy of various metrics in recommending the true number of clusters. We use the true parameters for the three simulation studies discussed in Section 3 to generate data for true G=2 , G=3 , G=4 , respectively, and we fit models with varying g∈{2,3,4,5} . We consider a sample size of n=1000 and a censoring rate of r=0.25 . Table 13 reports the information criteria (AIC, BIC, and ICL), the significance of classification metrics (Entropy and Avg Diag), and the *P*-value of the score test, averaged over 1000 samples. Table 12 reports the relative frequency of times the information criteria and the classification significance criteria. Assuming the score test is valid (i.e. we do not reject the null hypothesis of conditional independence) these are the metrics are used to determine the number of clusters in JLCMs.^4^ For the information criteria, naturally, the minimum value represents the optimal number of clusters, and the maximum value of the classification significance criteria is considered for the optimal number of clusters.

**Table 12.** Cluster selection frequency over 1000 simulations.

			Selection frequency				
Model	True G	Fitted G	AIC	BIC	ICL	Entropy	Avg Diag
JLCMM	2	**2**	42.7%	42.7%	42.4%	27.5%	58.0%
		3	34.5%	34.5%	34.5%	22.3%	42.0%
		4	18.9%	18.9%	19.1%	39.7%	0.0%
		5	4.0%	4.0%	4.0%	10.4%	0.0%
	3	2	39.7%	40.0%	40.0%	43.3%	49.6%
		**3**	35.9%	37.9%	37.9%	39.0%	40.3%
		4	14.1%	12.8%	12.8%	12.3%	6.7%
		5	10.3%	9.2%	9.2%	5.4%	3.4%
	4	2	7.9%	7.9%	7.9%	7.9%	72.6%
		3	21.9%	21.9%	21.9%	32.0%	27.4%
		**4**	50.0%	50.0%	50.0%	24.4%	0.0%
		5	20.2%	20.2%	20.2%	35.7%	0.0%
NN-JLCM	2	**2**	66.0%	94.4%	94.4%	94.4%	95.9%
		3	17.1%	4.6%	4.4%	3.8%	3.7%
		4	10.7%	1.0%	1.0%	1.2%	0.4%
		5	6.2%	0.0%	0.2%	0.6%	0.0%
	3	2	26.2%	26.4%	26.6%	61.0%	74.0%
		**3**	37.6%	45.8%	54.0%	30.2%	26.0%
		4	20.4%	24.6%	15.6%	5.2%	0.0%
		5	15.8%	3.2%	3.8%	3.6%	0.0%
	4	2	0.0%	0.0%	0.0%	0.0%	10.1%
		3	8.5%	11.1%	11.1%	22.5%	33.8%
		**4**	62.8%	79.9%	79.9%	73.8%	56.1%
		5	28.8%	9.1%	9.1%	3.6%	0.0%

JLCMM: joint latent class mixed model; NN-JLCM: neural-network joint latent class model; AIC: Akaike information criterion; BIC: Bayesian information criterion; ICL: integrated classification likelihood.

**Table 13.** Average simulation metrics by model and true G .

			Average metrics					
Model	True G	Fitted G	AIC	BIC	ICL	Entropy	Avg Diag	*P*-value
JLCMM	2	**2**	69056.6	69115.5	69398.6	0.591	0.908	– ${}^{a}$
		3	69835.8	69924.2	70561.2	0.420	0.618	– ${}^{a}$
		4	70358.6	70476.4	71024.9	0.604	— ${}^{b}$	– ${}^{a}$
		5	70815.1	70962.3	71654.5	0.570	— ${}^{b}$	– ${}^{a}$
	3	2	30865.0	30938.6	30961.7	0.967	0.982	0.055
		**3**	29861.7	29969.6	29982.3	0.988	0.991	0.159
		4	29612.9	29755.2	29791.3	0.974	0.963	0.330
		5	29445.0	29621.7	29678.3	0.965	0.950	0.405
	4	2	52873.3	52966.5	53168.1	0.709	0.913	0.309
		3	48970.6	49098.2	49105.6	0.993	0.839	0.329
		**4**	46139.8	46301.8	46340.9	0.972	– ${}^{b}$	0.496
		5	47082.7	47279.0	47279.1	1.000 ${}^{b}$	– ${}^{b}$	0.502
NN-JLCM	2	**2**	51063.6	51112.7	51114.2	0.998	0.999	0.520
		3	51036.8	51105.5	51327.3	0.798	0.795	0.519
		4	51035.5	51123.9	51513.6	0.719	0.697	0.523
		5	51050.2	51158.1	51699.9	0.663	0.620	0.522
	3	2	30374.7	30443.4	30502.9	0.914	0.971	0.457
		**3**	29337.5	29430.7	29494.8	0.942	0.922	0.465
		4	29304.4	29422.2	29533.4	0.920	0.854	0.463
		5	29259.1	29401.5	29532.1	0.919	0.828	0.459
	4	2	45485.0	45583.2	45613.8	0.956	0.988	0.302
		3	44549.0	44671.7	44696.9	0.977	0.990	0.331
		**4**	43833.0	43980.2	44027.6	0.966	0.966	0.498
		5	43956.0	44127.8	44244.3	0.928	0.872	0.506

JLCMM: joint latent class mixed model; NN-JLCM: neural-network joint latent class model; AIC: Akaike information criterion; BIC: Bayesian information criterion; ICL: integrated classification likelihood.

${}^{a}$ Score test *P*-values unavailable for JLCMM under True G=2 due to non-convergence of the score test computation.

${}^{b}$ Missing values for average diagonal arise from computational issues in the JLCM model fits.

The cluster selection frequency table in Table 12 reveals a clear distinction in model selection consistency between JLCMM and NN-JLCM. When the true number of clusters is 2, NN-JLCM demonstrates strong recovery across all information criteria, with BIC, ICL, entropy, and Avg Diag all selecting the correct model over 94% of the time. For JLCMM, AIC, and BIC select the true model less than half the time, and entropy selects G=4 most frequently (39.7%).

For G=4 , NN-JLCM’s BIC and ICL correctly identify the true model nearly 80% of the time, while JLCMM achieves only 50%. Notably, Avg Diag performs poorly for both models when true G exceeds 2, often favouring simpler models–selecting G=2 over 70% of the time for JLCMM when the true G is 4. It is speculated for G=3 that the complex covariance parameterization with the proportionality constraint on the covariances between groups, this could be contributing to the lower recovery rates with the NN-JLCM. The reason for this is that G=3 is the only simulation study which considered such a parameterization, whereas G=2 and G=4 do not. However, this particular behaviour requires further study.

For JLCMM with true G=2 , the information criteria values are relatively close across candidate models, explaining the indecisive selection frequencies. The entropy values are also low, indicating poor class separation and thus possibly unreliable entropy-based selection. NN-JLCM, however, achieves near-perfect entropy (0.998) at the true G=2 , which explains its superior selection performance.

The *P*-values hover around 0.5 for NN-JLCM across most scenarios, regardless of the fitted G . In addition, the *P*-values for the JLCMM are never <0.055, which means the null hypothesis is comfortably rejected at most 5% significance for all fitted cases. This is consistent with models satisfying conditional independence. JLCMM’s missing *P*-values for true G=2 indicate convergence issues, corroborating the erratic selection behaviour observed.

Overall, the results suggest that NN-JLCM provides more reliable model selection metrics, particularly in scenarios with well-separated classes, while JLCMM struggles to identify the correct number of clusters due to poor class separation and convergence issues.

### 4.4. Similar parameters (cluster overlap)

To assess the robustness of the NN-JLCM under weak class separation, we consider a two-component model in which the class-specific parameters are deliberately chosen to be close in value. The longitudinal submodel follows the same specification as in the previous scenario, with class-specific fixed effects $\beta_{1}=(170,2{)}^{⊺}$ and $\beta_{2}=(165,4{)}^{⊺}$ , a shared random effects covariance with Cholesky factor $\tau_{11}=7.746$ , and residual standard deviation $\sigma_{\varepsilon }=7.746$ . The survival submodel assumes class-specific Weibull distributions with shape parameters κ=(4,6) and scale parameters λ=(2,4) . Right-censoring times follow a Weibull distribution with shape $\tilde{\kappa }=2$ and scale $\tilde{\lambda }=10$ , yielding a censoring rate of ∼ 30%. The mixing proportions follow the same non-standard functional form as described in Scenario I in equation (51). The proximity of the class-specific parameters creates substantial overlap in both the longitudinal trajectories and survival curves, making class discrimination challenging.

Table 14 presents the parameter estimates, ABs, and mean squared errors for the NN-JLCM and JLCM under the cluster overlap scenario. The NN-JLCM recovers the true parameter values with high accuracy across all sample sizes, with ABs and MSEs decreasing consistently as n increases. By n=1000 , the fixed effects estimates are within 0.05 of their true values, and the Weibull parameters are similarly well-recovered. By contrast, the JLCM does not perform as well. The fixed effect estimates for the JLCM are biased with higher biases and MSEs in comparison to the NN-JLCM across the majority of estimates. Just as observed previously, the JLCM mixing proportion bias remains largely fixed regardless of sample size.

**Table 14.** Comparison of parameter estimates, absolute biases, and MSEs across methods.

			NN-JLCM			JLCM		
Sample size	Parameter	Truth	Estimate	AB	MSE	Estimate	AB	MSE
n=100	$\pi_{1}(x)$	–	–	0.1550	0.0408	–	0.3910	0.2140
	$\pi_{2}(x)$	–	–	0.1550	0.0408	–	0.3910	0.2140
	σ	7.746	7.7148	0.0312	0.0627	7.7774	0.0314	0.0592
	$d_{11}$	7.746	7.4366	0.3094	0.7740	7.2186	0.5274	9.7519
	$\beta_{11}$	170.00	170.3809	0.3809	5.6683	203.3573	33.35728	2823.7023
	$\beta_{12}$	165.00	164.6685	0.3315	3.6189	121.9249	43.0751	3576.1505
	$\beta_{21}$	2.00	1.9662	0.0338	2.2253	2.2955	0.2955	16.0705
	$\beta_{22}$	4.00	3.9460	0.0540	1.2819	4.5629	0.5629	17.9122
	$\kappa_{1}$	4.00	3.7474	0.2526	0.5825	4.6246	0.6246	1.4841
	$\kappa_{2}$	6.00	6.0395	0.0395	0.1145	862.9048	856.9048	$3.61\times 10^{8}$
	$\lambda_{1}$	2.00	2.1834	0.1834	0.1819	2.1720	0.1720	0.3492
	$\lambda_{2}$	4.00	4.3611	0.3611	0.6752	2.8211	1.1789	1.5533
n=500	$\pi_{1}(x)$	–	–	0.0787	0.0104	–	0.3910	0.2130
	$\pi_{2}(x)$	–	–	0.0787	0.0104	–	0.3910	0.2130
	σ	7.746	7.7257	0.0203	0.0128	7.7628	0.0168	0.0117
	$d_{11}$	7.746	7.6945	0.0515	0.0972	7.2255	0.5205	10.8494
	$\beta_{11}$	170.00	170.1429	0.1429	1.1089	202.1930	32.1930	2619.4567
	$\beta_{12}$	165.00	164.9739	0.0261	0.5007	123.7126	41.2874	3312.2785
	$\beta_{21}$	2.00	1.9194	0.0806	0.3479	1.9652	0.0348	22.2865
	$\beta_{22}$	4.00	4.0233	0.0233	0.1432	5.1437	1.1437	29.7385
	$\kappa_{1}$	4.00	3.9226	0.0774	0.1213	4.8788	0.8788	1.3980
	$\kappa_{2}$	6.00	5.9989	0.0011	0.0235	14.9489	8.9489	3786.46
	$\lambda_{1}$	2.00	2.0284	0.0284	0.0206	2.1545	0.1545	0.2194
	$\lambda_{2}$	4.00	4.0739	0.0739	0.1116	2.7166	1.2834	1.7091
n=1000	$\pi_{1}(x)$	–	–	0.0571	0.0055	–	0.3900	0.2130
	$\pi_{2}(x)$	–	–	0.0571	0.0055	–	0.3900	0.2130
	σ	7.746	7.7427	0.0033	0.0062	7.7735	0.0275	0.0066
	$d_{11}$	7.746	7.7142	0.0318	0.0506	6.8552	0.8908	16.8069
	$\beta_{11}$	170.00	170.0541	0.0541	0.4708	202.7156	32.7487	2589.1244
	$\beta_{12}$	165.00	164.9529	0.0471	0.2190	125.9031	38.9071	3043.1157
	$\beta_{21}$	2.00	2.0001	0.0001	0.1431	1.3153	0.6947	15.1316
	$\beta_{22}$	4.00	4.0048	0.0048	0.0618	5.3378	1.3923	17.8500
	$\kappa_{1}$	4.00	3.9721	0.0279	0.0583	4.9229	0.9229	1.4812
	$\kappa_{2}$	6.00	6.0140	0.0140	0.0113	27312.92	27306.92	$4.56\times 10^{11}$
	$\lambda_{1}$	2.00	2.0214	0.0214	0.0102	2.0574	0.0574	0.2848
	$\lambda_{2}$	4.00	4.0550	0.0550	0.0528	2.7098	1.2902	1.7235

MSEs: mean squared errors; NN-JLCM: neural-network joint latent class model; JLCM: joint latent class model; AB: absolute bias.

In addition, the mixing proportion bias is larger here for JLCM at around 0.39 in comparison with 0.28 under the same mixing proportion structure in Scenario I. This shows that the logistic class-membership model is structurally unable to recover the true mixing proportions, which are non-monotonic functions, and the bias is compounded when cluster overlap is substantial. The NN-JLCM, by contrast, reduces mixing proportion bias from 0.155 at n=100 to 0.057 at n=1000 , demonstrating the advantage of a flexible neural network specification for the class-membership model. However, the cluster overlap also leads to slightly higher bias and MSE for the NN-JLCM compared to the previous scenario, which is expected given the increased difficulty of class discrimination.

Table 15 reports the goodness-of-fit and score test statistics. The NN-JLCM achieves substantially lower CE compared to the JLCM, indicating that the JLCM assigns subjects to classes at little better than chance when clusters overlap. This misclassification propagates to all predictive metrics. The JLCM produces in-sample longitudinal MSE values in the thousands, whereas the NN-JLCM achieves reasonable MSE scores. The IBSs follow a similar pattern, with the NN-JLCM yielding lower values both in-sample and out-of-sample.

**Table 15.** Comparison of goodness-of-fit and score test metrics across methods.

			IBS		MSE Y		Score test		
n	Method	CE	In	Out	In	Out	Statistic	P -value	Null rejection rate
100	NN-JLCM	0.2364	0.0923	0.1375	51.0849	54.5025	0.8237	0.5404	0
	JLCM	0.6088	0.1251	0.1679	3711.65	125.334	–	–	–
500	NN-JLCM	0.1982	0.0888	0.1258	51.1912	51.3154	0.7691	0.5501	0
	JLCM	0.6082	0.1147	0.1542	3581.35	123.518	–	–	–
1000	NN-JLCM	0.1900	0.0862	0.1214	51.4400	51.1681	0.8043	0.5388	0
	JLCM	0.6086	0.1112	0.1600	3168.92	123.596	3.6339	0.1625	0

NN-JLCM: neural-network joint latent class model; JLCMM: joint latent class mixed model; CE: classification error; IBS: integrated Brier score; MSE: mean squared error.

The score test for conditional independence fails to reject the null for the NN-JLCM at all sample sizes (statistic ≈0.8 , P≈0.54 ), which is the correct outcome given that the data is generated from a conditional independence model. The JLCM score test is unavailable at n=100 and n=500 due to convergence failures, and at n=1000 also fails to reject ( P=0.16 ). Together, these results demonstrate that the NN-JLCM is more robust to cluster overlap under a NN-JLCM DGP than the standard JLCM, maintaining accurate parameter recovery and classification where the logistic specification of JLCM struggles significantly.

### 4.5. Synthetic data analysis

To investigate NN-JLCM’s ability to distinguish between data which conforms to the assumptions of conditional independence, we consider a mirrored approach. Here, data from an SREM is generated, and the various competing models are fit, and then data from a G=2 NN-JLCM is generated, and then competing models are fit. The IBS, MSE, and Score test statistics are reported for each fitted model in order to evaluate the goodness-of-fit and the ability of the score test to correctly reject the null hypothesis of conditional independence when it is violated, and fail to reject when it is satisfied.

Further, where the score test is not appropriate, such as the SREM, the Wald test of the association parameter α is used to evaluate whether the model correctly identifies the presence of an association between the longitudinal and survival processes. Naturally, biases and MSEs are less interpretable to this end, and so, in addition to reasons of brevity, we report goodness-of-fit statistics only. However, tables of estimates can be requested from the corresponding author.

#### 4.5.1. Generating from an SREM

The settings of this synthetic data analysis are inspired by the primary biliary cholangitis (PBC) dataset from the Mayo Clinic, which has been extensively used in survival analysis literature.^51^ The PBC dataset contains longitudinal measurements of serum bilirubin, a biomarker indicative of liver function, alongside time-to-event outcomes for patients with PBC.

We generate data from an SREM to evaluate the score test of conditional independence under a known violation of the JLCM assumption. We briefly discuss the SREM parameterization here but interested readers are directed to Rizopoulos^7^ and Papageorgiou et al.^12^ for more information. The longitudinal process follows a linear mixed-effects model

(66) $$y_{ij}=(\beta_{0}+b_{0i})+(\beta_{1}+b_{1i})t_{ij}+\varepsilon_{ij}$$

with fixed effects $\beta_{0}=0.507$ and $\beta_{1}=0.173$ , residual error $\varepsilon_{ij}∼N(0,\sigma_{\varepsilon }^{2})$ with $\sigma_{\varepsilon }=0.344$ , and random effects $b_{i}=(b_{0i},b_{1i}{)}^{⊺}∼N(0,D)$ , where

(67) $$\begin{array}{r} D=\left(\begin{array}{cc} 0.985 & 0.055\\ 0.055 & 0.031 \end{array}\right) \end{array}$$

The survival process is specified as a Weibull proportional hazards model with the hazard function for subject i at time t given by the following equation:

(68) $$h_{i}(t)=\kappa t^{\kappa -1}\exp\;\left(\gamma_{0}+\gamma_{1}\;{\mathrm{age}}_{i}+\alpha \;\eta_{i}(t)\right)$$

where $\eta_{i}(t)=(\beta_{0}+b_{0i})+(\beta_{1}+b_{1i})t$ is the true underlying longitudinal trajectory, κ=exp(0.984)=2.674 is the Weibull shape parameter, $\gamma_{0}=-15.774$ is the survival intercept, $\gamma_{1}=0.073$ is the age effect, and α=1.384 is the association parameter linking the longitudinal and survival processes. Baseline age is generated as ${\mathrm{age}}_{i}∼N(50,10^{2})$ . Survival times are obtained by inverting the cumulative hazard function via numerical root-finding (see Brent,^52^ Brilleman et al.,^53^ and Crowther and Lambert^54^ for more details), with administrative censoring at 15 years. We consider sample sizes n∈{100,500,1000} with 1000 Monte Carlo replications per configuration.

The NN-JLCM and JLCM specifications are the same for the longitudinal and survival submodels with G=2 . Thus, we consider a linear mixed-effects model with class-varying fixed effects and random intercept and slope for the longitudinal submodel, and a Weibull proportional hazards model for the survival submodel with class-specific baseline hazards. Additionally, age is included as a class-common covariate in the survival submodel. The class-membership model is specified as a function of baseline age only. The SREM is fit according to the same specifications as the DGP. The JLCT model is fit with the same longitudinal, survival, and class-membership submodel specifications as the JLCM.

##### Results

Table 16 presents the goodness-of-fit statistics for the NN-JLCM, JLCMM, SREM, and JLCT under the SREM DGP. As expected, the SREM consistently achieves the lowest IBS values across all sample sizes, since the fitted model coincides with the true data-generating mechanism. The NN-JLCM and JLCMM yield comparable in-sample IBS values to the SREM, though both exhibit slightly higher out-of-sample IBS, with the JLCMM producing marginally larger values than the NN-JLCM. The JLCT exhibits the poorest predictive performance, with IBS values approximately double those of the other models and substantially inflated MSE values, reflecting the limitations of the tree-based partitioning approach in capturing the smooth, continuous heterogeneity induced by the shared random effects structure. The most notable distinction between the two latent class approaches appears in the longitudinal prediction accuracy. NN-JLCM achieves MSE values matching the SREM, whereas the JLCMM yields substantially inflated MSE values ranging from 5.10 to 6.85. This discrepancy reflects the greater flexibility of the neural network-based mixing proportions in capturing the class-membership structure, which in turn improves the allocation of subjects to their correct longitudinal trajectories.

**Table 16.** Goodness-of-fit statistics across sample sizes (SREM DGP, G=2 ).

		IBS		MSE Y		Test of model assumptions		
*n*	Method	In	Out	In	Out	Statistic	P -value	Null rejection rate
100	NN-JLCM	0.0634	0.0787	0.1026	0.0040	35.197	$6.45\times 10^{-6}$	1
	JLCMM	0.0641	0.0846	6.8547	6.3869	35.364	$9.13\times 10^{-6}$	1
	SREM	0.0634	0.0642	0.1025	0.1027	9.443 ${}^{a}$	$4.47\times 10^{-18}$	1
	JLCT	0.1202	0.1202	2.9150	2.9880	–	–	–
500	NN-JLCM	0.0621	0.0736	0.1027	0.0037	175.029	$2.38\times 10^{-31}$	1
	JLCMM	0.0622	0.0829	5.2983	5.1993	174.989	$1.23\times 10^{-30}$	1
	SREM	0.0601	0.0602	0.1030	0.1030	21.299 ${}^{a}$	$1.54\times 10^{-90}$	1
	JLCT	0.1241	0.1241	2.9680	2.9810	–	–	–
1000	NN-JLCM	0.0620	0.0731	0.1027	0.0037	348.387	$5.98\times 10^{-60}$	1
	JLCMM	0.0620	0.0828	5.2815	5.1008	346.925	$1.05\times 10^{-59}$	1
	SREM	0.0598	0.0598	0.1028	0.1028	30.189 ${}^{a}$	$4.97\times 10^{-188}$	1
	JLCT	0.1243	0.1243	2.9760	2.9820	–	–	–

SREM: shared random effects model; DGP: data-generating process; NN-JLCM: neural-network joint latent class model; JLCMM: joint latent class mixed model; JLCT: joint latent class tree; IBS: integrated Brier score; MSE: mean squared error.

${}^{a}$ For the SREM, the reported statistic is the Wald test for the association parameter α=0 , rather than the score test of conditional independence, which is not applicable under the shared random effects framework.

The score test of conditional independence correctly rejects the null hypothesis for both the NN-JLCM and JLCMM at all sample sizes, with test statistics increasing proportionally with n , consistent with the expected behaviour under a true violation of conditional independence. The SREM Wald test for the association parameter α similarly rejects the null of no association at all sample sizes, confirming that the shared random effects mechanism is detected by both testing frameworks. The null rejection rate of 1 for the NN-JLCM and JLCMM indicates that the score test rejects conditional independence in every Monte Carlo replication, while the rejection rate of 1 for the SREM indicates that the Wald test for α=0 rejects in every replication. Together, these results demonstrate that the score test implemented in the NN-JLCM is well-calibrated and has sufficient power to detect departures from conditional independence when the true DGP is an SREM. We report no hypothesis test for JLCT as the test done in JLCT is only a measure for splitting and not a reflection of the true underlying association of the data.^17^

#### 4.5.2. Generating from a NN-JLCM

The settings of this simulation scenario are also inspired by the PBC dataset.^51^ We assume that the true survival times $T^{*}$ are governed by a two-component mixture Weibull distribution with scale parameter $\lambda_{g}$ and shape parameter $\kappa_{g}$ for g=1,2 . The survival probability conditional on latent class membership is computed as follows:

(69) $$S(t){|}_{\left(c_{i}=g\right)}=\exp\;\left(-{\left(\frac{t}{\lambda_{g}}\right)}^{\kappa_{g}}\right)$$

The true values for the Weibull parameters are $\kappa_{1}=12.76$ , $\kappa_{2}=29.68$ , $\lambda_{1}=27.91$ , and $\lambda_{2}=37.89$ .

We assume that the right-censoring times C follow a Weibull distribution with parameters $\tilde{\lambda }$ and $\tilde{\kappa }$ , with values chosen to achieve a desired censoring rate r . We consider a censoring rate that would be similar to the true PBC dataset, which is around 33% censored observations. Therefore, the censoring distribution parameters are given as $\tilde{\kappa }=0.9$ and $\tilde{\lambda }=100$ .

To simulate the longitudinal profiles reflecting serum bilirubin trajectories, we consider a class-specific linear mixed-effects model with random intercept and random slope

(70) $$y_{ij}{|}_{\left(c_{i}=g\right)}=\beta_{1g}+\beta_{2g}t_{ij}+b_{0i}+b_{1i}t_{ij}+\varepsilon_{ij}$$

where the random effects follow a bivariate normal distribution

(71) $$\begin{array}{r} b_{i}=\left(\begin{array}{c} b_{0i}\\ b_{1i} \end{array}\right)∼N\left(0,D\right),\;D=\left(\begin{array}{cc} 0.68 & -0.02\\ -0.02 & 0.12 \end{array}\right) \end{array}$$

and $\varepsilon_{ij}∼N(0,\sigma_{\varepsilon }^{2})$ with $\sigma_{\varepsilon }^{2}=0.35$ .

The class-specific fixed effects are organized into vectors $\beta_{g}=(\beta_{1g},\beta_{2g}{)}^{⊺}$ , with the full fixed-effects matrix given by the following equation:

(72) $$\begin{array}{r} \beta =\left(\begin{array}{cc} \beta_{11} & \beta_{21}\\ \beta_{12} & \beta_{22} \end{array}\right)=\left(\begin{array}{cc} 1.45 & 0.35\\ -0.06 & 0.09 \end{array}\right) \end{array}$$

To investigate the performance of the methods under non-standard mixing proportion specifications, we adopt a functional form for the class-membership probabilities inspired by the following equation^20^:

(73) $$\pi_{1}(x_{i})=(x_{i}-0.5{)}^{2}\;\text{and}\;\pi_{2}(x_{i})=1-\pi_{1}(x_{i})$$

where $x_{i}$ is generated from a uniform distribution on the interval (0,1) . This quadratic specification produces a non-monotonic relationship between the covariate and class membership probability, which violates the assumptions of standard logistic regression-based mixing proportion models.

**Figure 6.**
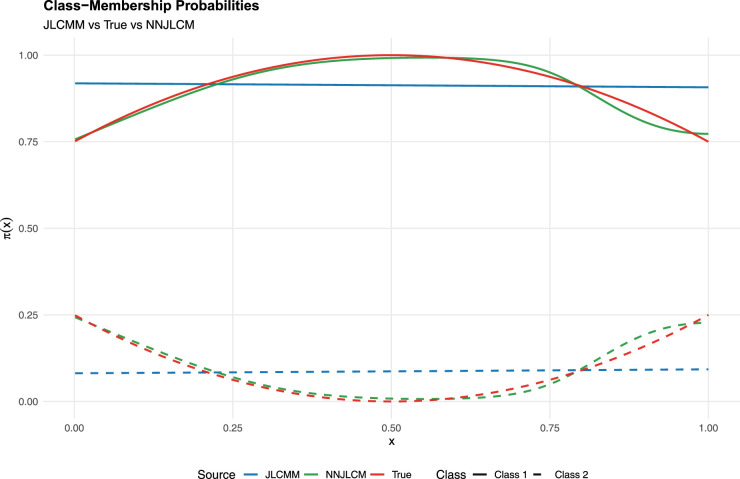
Mixing proportion comparisons for the two-component model under study.

We consider three sample sizes: n∈{100,500,1000} , to evaluate the performance of the estimators across small, moderate, and large sample settings. For each configuration, we conduct 1000 Monte Carlo replications and compare our proposed NN-JLCM against the JLCM, the JLCT, and the SREM. Performance is assessed via the average estimate, AB, and MSE for each parameter. Additionally, we assess the goodness-of-fit by reporting the score test of conditional independence as well as the *P*-values of the significance of the association parameter in the SREM. The goodness-of-fit and predictive performance statistics are reported in Table 17. An example of the fit for the mixing proportions for NN-JLCM and JLCMM against the true mixing proportions is visualized in Figure 6.

**Table 17.** Comparison of goodness-of-fit metrics across methods.

			IBS		MSE Y		Test of model assumptions		
*n*	Method	CE	In	Out	In	Out	Statistic	P -value	Null rejection rate
100	NN-JLCM	0.0306	0.0236	0.0292	0.0943	0.0936	1.5161	0.5569	0
	JLCMM	0.0131	0.0259	0.0318	0.7735	1.1681	2.0787	0.4795	0.0533
	JLCT	0.4851	0.0343	0.0343	1.4100	1.4500	–	–	–
	SREM	–	0.0397	0.0431	0.0938	0.0940	9.43776 ${}^{a}$	0.2380	0.2910
500	NN-JLCM	0.0066	0.0223	0.0268	0.0938	0.0932	1.2167	0.6199	0
	JLCMM	0.0072	0.0229	0.0281	0.7641	1.1371	1.9251	0.4987	0.0368
	JLCT	0.4119	0.0381	0.0380	1.4300	1.4400	–	–	–
	SREM	–	0.0387	0.0417	0.0935	0.0936	21.2869 ${}^{a}$	0.0139	0.9210
1000	NN-JLCM	0.0064	0.0221	0.0262	0.0938	0.0932	1.0845	0.6480	0
	JLCMM	0.0071	0.0225	0.0275	0.7637	1.1431	1.9571	0.5048	0.0511
	JLCT	0.4121	0.0375	0.0376	1.4100	1.4100	–	–	–
	SREM	–	0.0385	0.0411	0.0934	0.0934	30.1524 ${}^{a}$	0.0005	0.9990

NN-JLCM: neural-network joint latent class model; JLCMM: joint latent class mixed model; JLCT: joint latent class tree; SREM: shared random effects model; CE: classification error; IBS: integrated Brier score; MSE: mean squared error.

${}^{a}$ For the SREM, the reported statistic is the Wald test for the association parameter α=0 , rather than the score test of conditional independence, which is not applicable under the shared random effects framework.

##### Results

The comparison between the four models is revealed in Table 17. In terms of goodness-of-fit, NN-JLCM consistently achieves the lowest in-sample and out-of-sample IBS across all sample sizes, with JLCM producing comparable but slightly higher values, whereas JLCT and SREM lag noticeably behind. The MSE of the longitudinal outcome shows that NN-JLCM and SREM yield nearly identical and substantially lower MSE values, while JLCM produces considerably inflated MSE values, particularly out-of-sample (e.g. 1.1681 at n=100 ), and JLCT performs worst with MSE values exceeding 1.4 across all settings. The CE for NN-JLCM decreases as n increases, dropping from 0.0306 at n=100 to 0.0064 at n=1000 , whereas JLCM maintains the same CE across sample sizes, again revealing the JLCMM’s inability to capture the underlying data structure when the class-membership model does not conform to the logistic regression assumptions.

Regarding the score test, both NN-JLCM and JLCM yield non-significant *P*-values across all sample sizes, indicating adequate model specification; however, NN-JLCM achieves a null rejection rate of 1.0 in all cases compared to JLCM’s rates around 0.95, suggesting that NN-JLCM more reliably satisfies the distributional assumptions of the score test for this simulation. Interestingly, the SREM’s Wald test for α=0 yields significant *P*-values at n=500 and n=1000 despite generating from a model with α=0 . This may indicate that the SREM is more sensitive to model misspecification in this scenario, or that the test for α does not have sufficient power to detect the true null under this setting. Again, there is no hypothesis test to report for JLCT as the LRT test used in the splitting routine is not appropriate here.

## 5. Application

In this section, we demonstrate the practical use of the proposed model on a real dataset and compare our findings to established models.

### 5.1. PAQUID data description

The PAQUID dataset consists of a random subsample of 500 subjects from the study of a French cohort about dementia and Alzheimer’s incidence rates.^27^ The dataset is the benchmark dataset used for both the jlctree^17^ and lcmm^55^ packages for JLCM, and is available for download from the latter. Repeated measurements of the mini-mental state examination, stored as *MMSE*, Isaac’s Set Test, stored as *IST* and the Benton visual retention test, stored as *BVRT*, were captured. Additionally, a Center for Epidemiologic Studies Depression Scale (CESD) test was administered, stored as *CESD*, and a rating of physical dependency, stored as *HIER*, were recorded over a 20-year period.

Alongside this was the recording of age (*age*), dementia diagnosis status, *dem*, and age at dementia *agedem*. Baseline covariates recorded at enrolment of the cohort are education (*CEP*), sex (*male*), and age at enrolment (*age_init*). *MMSE* tends to be very skewed in terms of distribution, so the variable is normalized when used in analysis in joint modelling. Age is also replaced with *age_65*, which is determined by $\frac{\text{age}-65}{10}$ . Centreing age by 65 allows for clearer interpretations, and dividing by 10 allows for better numerical stability in estimations.^9^ The objective here is to jointly model the repeated measurements of the normalized *MMSE* and the event times for dementia occurrence.

**Figure 7.**
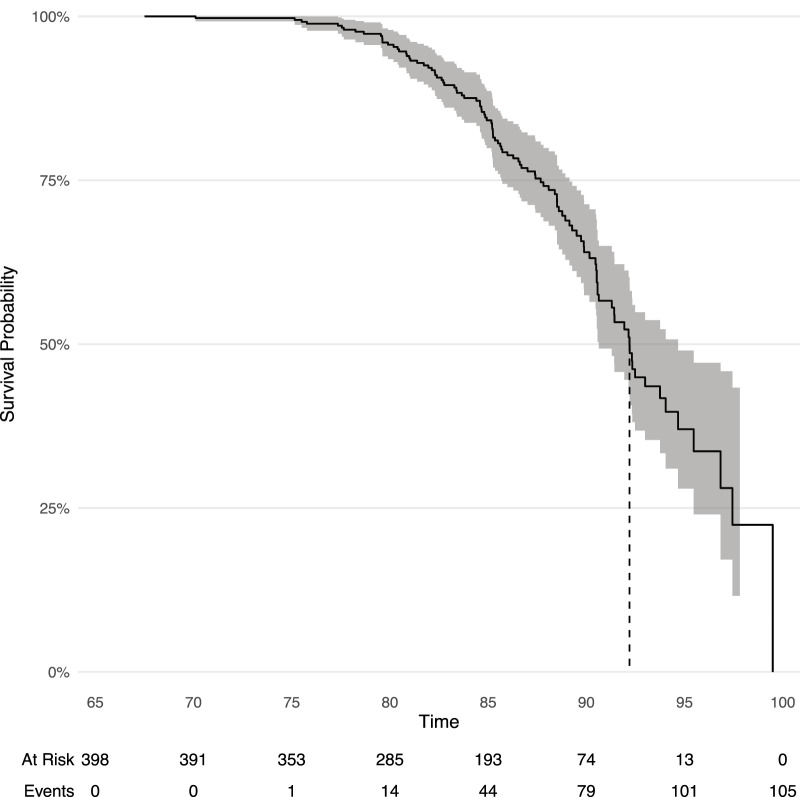
Kaplan-Meier survival curve for the PAQUID dataset.

We plotted the Kaplan-Meier survival curve in Figure 7 along with the risk table. Based on the data, 26% of individuals were diagnosed with dementia at the end of the study. Conversely, there is a very high rate of censoring in the dataset, with a large number of censored times occurring between t=70 and t=85 . The median event time is t=92.2 , denoted by the dashed line on the plot.

**Figure 8.**
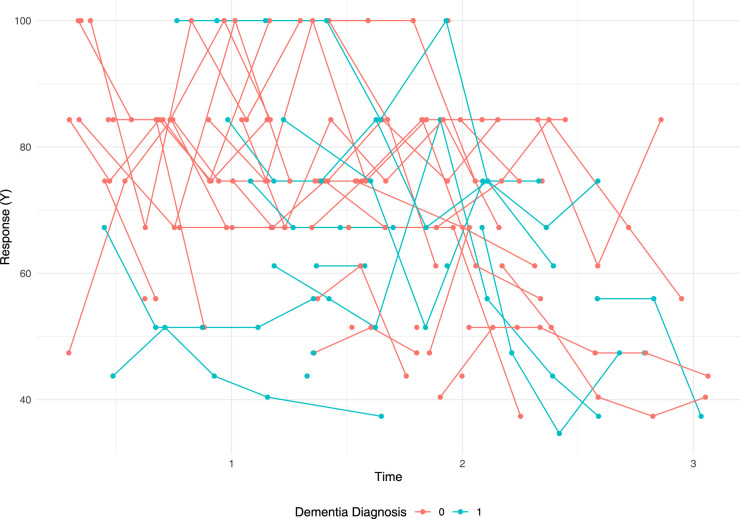
Trajectories for the normalized mini-mental state examination (MMSE) of 50 randomly sampled subjects from the PAQUID study.

Additionally, we plotted the trajectories of the normalized MMSE scores for the PAQUID dataset, where highlighted individuals correspond to a dementia diagnosis (0 indicating no diagnosis and 1 indicating diagnosis). There is not really a clear separation in the trajectories that distinguishes patients with and without dementia for this score, which may suggest the heterogeneity in the population is not directly distinguishable from the plot of the longitudinal profiles (Figure 8).

### 5.2. Model formulation and data preparation

We consider an 80:20 training-testing split to consider both in-sample and out-of-sample performance of all the models considered. For the class-membership covariates for JLCM, NN-JLCM and JLCT model, we let $v_{i}=({\text{CEP}}_{i},{\text{male}}_{i})$ . For the longitudinal submodel of all methods, let $X_{i}=(1,age\_65_{i},age\_65_{i}^{2})=Z_{i}$ and ${\tilde{X}}_{i}=({\text{CEP}}_{i})$ . And finally, for the survival submodel of all methods, we assume a proportional Weibull baseline hazards model with ${\tilde{w}}_{i}=v_{i}$ and no class-varying baseline covariates.

**Table 18.** Model comparison results.

								IBS		MSE	
Method	*G*	ℓ(θ)	BIC	AIC	*E*	ICL	Avg Diag	In	Out	In	Out
NN-JLCM	2	−6668.167	13456.06	13376.33	0.5294	13601.20	0.8484	0.0541	0.0529	72.672	67.491
NN-JLCM	3	−6660.954	13471.57	13371.91	0.6759	13612.18	0.8478	0.0512	0.0507	73.319	68.687
NN-JLCM	4	−6654.183	13487.96	13368.37	0.4823	13792.08	0.7000	0.0476	0.0480	72.686	68.848
JLCMM	2	−6648.479	13434.65	13342.96	0.5078	13585.49	0.8431	0.0750	0.0560	72.521	175.354
JLCMM	3	−6640.856	13467.29	13343.71	0.7097	13604.15	0.8587	0.0738	0.0563	73.069	168.726
JLCMM	4	−6643.432	13520.34	13364.86	0.6565	13724.86	–	0.0827	0.1004	72.199	146.877
SREM	–	−6605.922	13243.84	13307.63	–	–	–	0.1284	0.1946	74.50037	68.040
JLCT	3	–	–	–	–	–	–	0.1164	0.1266	73.4012	208.9867

NN-JLCM: neural-network joint latent class model; JLCMM: joint latent class mixed model; SREM: shared random effects model; JLCT: joint latent class tree; AIC: Akaike information criterion; BIC: Bayesian information criterion; ICL: integrated classification likelihood; IBS: integrated Brier score; MSE: mean squared error.

**Figure 9.**
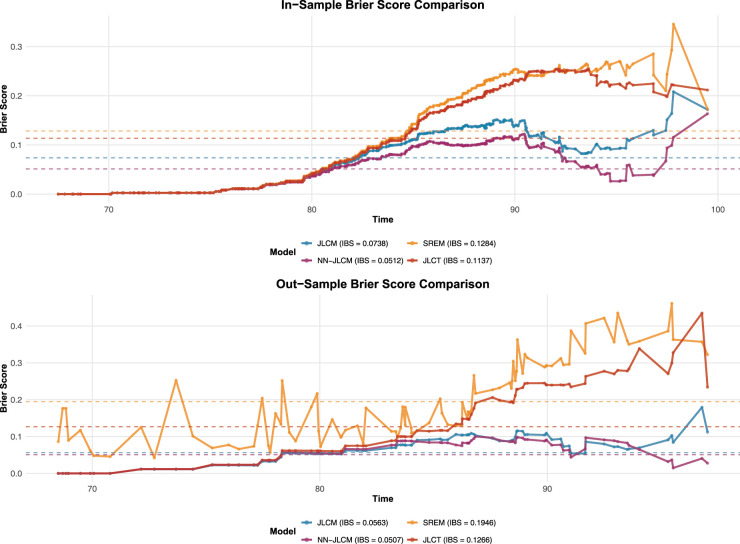
Comparison of Brier scores of survival submodels with integrated Brier score (IBS) indicated in dashes.

### 5.3. Results discussion

We present the performance metrics of the models assessed in Table 18. Again, to determine the ideal number of clusters, we must consider a compromise among a collection of metrics, as it is not so straightforward. If we consider the BIC and ICLs only (i.e. just the information criteria), the ideal model is a G=2 model. However, this does not factor in classification meaningfulness nor the predictive abilities of either model, as these measures are only heuristic in nature. Furthermore, if we consider the best model solely on AIC and BIC without considering classification and prediction, the ideal model is then an SREM.

Moving to the classification abilities of the models, the models yielding the best entropy values and the highest average diagonal responsibilities (in the columns entropy and Avg. Diag., respectively) are the three-component models. The probabilities are not computed for the four-class model for JLCMM, as it could not converge after 250 iterations. This also implies the score test could not be computed for that model. Finally, if we consider the predictive abilities of the models, where we can also compare with the JLCT model and the SREM, we can see that the model with the best survival and longitudinal predictive ability for NN-JLCM is one with g=4 clusters, and g=3 clusters for JLCMM. However, the g=4 classification ability of NN-JLCM is not as powerful as g=3 , which according to literature, along with the information criteria, are more important in terms of selection of the number of clusters. Therefore, we consider the g=3 model. Indeed, the optimal number of clusters determined from JLCT found g=3 to be the ideal number of clusters using the nonparametric tree-based approach, which also confirms the choice of g=3 . Both NN-JLCM and JLCM conform to the hypothesis of conditional independence for g=3 , yielding *P*-values >10%, implying at any reasonable level of significance, the null hypothesis of conditional independence is not rejected.

In the classical JLCM, all of the covariates in the multinomial logit for class membership were statistically insignificant (not reported), which would suggest that the linear parametric form does not capture the drivers of latent class assignment in this dataset. This is an issue as it effectively limits the JLCM to estimating constant class probabilities.

The NN-JLCM relaxes the linearity restriction and allows for nonlinear mappings from covariates to class probabilities. The results from Table 18 confirm that this additional flexibility improves both the longitudinal and survival predictions. The NN-JLCM showed improvements for both the out-of-sample and in-sample IBS, relative to all other models. To evaluate the goodness-of-fit of our selected models, the BSs were plotted for the entire range of event times in Figure 9. We can see that the BS for every model is about the same until $t_{i}=80$ , where significant divergence in performance is observed. From here, we can see that NN-JLCM remains the smallest across all models, while the SREM remains the largest. These results indicate that the NN-JLCM not only generalizes better to unseen data but also provides a closer fit to the observed survival outcomes.

In summary, the evidence from the PAQUID dataset suggests that the NN-JLCM provides more accurate predictions, clearer class separation, and more robust generalization. The classical JLCM, by contrast, was hindered by the lack of significance in the multinomial covariates for class membership, highlighting the benefits of using a neural network to model mixing proportions.

## 6. Conclusion and discussion

In this article, we proposed a NN-JLCM for modelling univariate longitudinal and time-to-event data using an EM-type algorithm to estimate the parameters by maximum likelihood. Extensive simulation studies under varying sample sizes and censoring rates confirmed the competitiveness and robustness of the new method in estimating the survival and longitudinal submodels. Further, simulations in Section 3 showed that if the true class-membership model does not conform to the assumptions of the traditional logistic regression model this can lead to severe biases in predicted survival and longitudinal times as well as poor class discrimination and class-predictions.

Additional studies of finite sample properties in Section 4 considered the efficacy of traditional metrics for latent class selection, a simulation showcasing performance under cluster overlap, and a synthetic data analysis, firstly, considering the effect of generating from an SREM and evaluating the NN-JLCM, JLCMM and the JLCT methods alongside the SREM, and then secondly considering the effect of generating from our proposed model and evaluating all the above competing methods. The purpose of this is to observe NN-JLCM’s behaviour when generating from data which does not conform to the assumptions of conditional independence and vice versa, evaluated through the lens of the score test of conditional independence.

The additional simulations provided useful insights, in that the information criteria and classification metrics under the assumption of a valid score test, adequately predict the true latent class when the class-membership model veers away from the assumptions of the logistic regression model. Further, cluster overlap has mild effects on the performance of NN-JLCM, with a higher CE relative to situations with better class separation. Finally, the synthetic data analysis simulation showed that NN-JLCM behaves as expected when the DGP comes from an SREM, providing an adequate fit in terms of IBS and MSE (comparable to JLCMM) but rejecting the assumption of conditional independence entirely under such a DGP. Similarly, when the DGP conforms to a JLCM, the assumption of conditional independence is held by both NN-JLCM and JLCM.

The application to the PAQUID dataset showcased that while the JLCMM provided an adequate fit in terms of parameters and predictions, the covariates of the multinomial regression were all insignificant and the NN-JLCM still outperformed the JLCM in survival predictions. Additionally, the JLCT model and SREMs were also considered; however, their performance was worse than either JLCM and NN-JLCM. Interestingly, Zhang and Simonoff^17^ performed a similar application to the one presented in this article; however, the authors considered a 10-fold cross-validation and evaluated the models only on test data from the cross-validation. Even so, the IBS and MSEs reported here suggest even better performance than what JLCT could achieve. The performance of the SREM is roughly in line with what was observed by Zhang and Simonoff,^17^ but to truly confirm this, a cross-validation of NN-JLCM could be investigated on a number of datasets to compare with JLCT.

One of the limitations of the proposed model is that by using a neural network in the class-membership model, we inherently lose explainability and ease of interpretation. Since we use an EM-type algorithm to estimate the parameters, convergence could be slow relative to the Marquardt algorithm implemented in lcmm. Finally, there is a more abundant suite of available tools for modelling from lcmm, which has not yet been developed for use with NN-JLCM, such as dynamic predictions.

From here, a potential avenue of future work that could be considered is extensions to multivariate longitudinal data and multivariate time-to-event data. Additionally, the use of semi-parametric specifications for the baseline hazard function could be considered, as well as extensions to other types of longitudinal outcomes such as binary and count data.

## Supplemental Material

sj-pdf-1-smm-10.1177_09622802261465334 - Supplemental material for Deep learning embedded latent class joint modelling of time-to-event and longitudinal data Supplemental material, sj-pdf-1-smm-10.1177_09622802261465334 for Deep learning embedded latent class joint modelling of time-to-event and longitudinal data by Tristan John Elers Harris, Najmeh Nakhaei Rad and Sphiwe Bonakele Skhosana in Statistical Methods in Medical Research
