# Supplementary material for: Deep learning embedded latent class joint modelling of time-to-event and longitudinal data
Source: Stat Methods Med Res. 2026 Jul 10;35(8):1746–84. doi: 10.1177/09622802261465334 (PMC13478454; doi:10.1177/09622802261465334)
Supplement: Supplementary file 1 [file sj-pdf-1-smm-10.1177_09622802261465334.pdf]

S1. Supplementary Materials

S1.1. Two component model

Table S1: Bias and MSE Results for NN-JLCM $G = 2$ and JLCMM Parameter Estimates across Sample Sizes ($B = 1000$ simulations, 25% censoring)

			**NN-JLCM**			**JLCMM**		
Sample Size	Parameter	Truth	Estimate	AB	MSE	Estimate	AB	MSE
n=100	$\pi_1(x)$	–	–	0.014	0.007	–	0.283	0.118
	$\pi_2(x)$	–	–	0.014	0.007	–	0.283	0.118
	σ_e	7.746	7.734	0.012	0.060	7.734	0.012	0.060
	β_{11}	170	169.974	0.026	1.745	169.976	0.024	1.741
	β_{12}	88	87.976	0.024	0.682	87.975	0.025	0.682
	β_{21}	100	99.977	0.023	1.092	99.976	0.024	1.091
	β_{22}	1.2	1.213	0.013	0.381	1.211	0.011	0.380
	σ_b	7.071	6.985	0.086	0.387	6.985	0.086	0.387
	κ_1	2	2.087	0.087	0.084	2.087	0.087	0.084
	κ_2	1.01	1.030	0.020	0.025	1.030	0.020	0.025
	λ_1	4.5	4.493	0.007	0.155	4.493	0.007	0.155
	λ_2	50	51.239	1.239	88.422	51.244	1.244	88.396
n=500	$\pi_1(x)$	–	–	0.014	0.002	–	0.287	0.111
	$\pi_2(x)$	–	–	0.014	0.002	–	0.287	0.111
	σ_e	7.746	7.738	0.008	0.013	7.738	0.008	0.013
	β_{11}	170	170.031	0.031	0.381	170.031	0.031	0.381
	β_{12}	88	87.992	0.008	0.133	87.992	0.008	0.133
	β_{21}	100	100.010	0.010	0.220	100.010	0.010	0.220
	β_{22}	1.2	1.209	0.009	0.076	1.209	0.009	0.076
	σ_b	7.071	7.060	0.011	0.071	7.060	0.011	0.071
	κ_1	2	2.016	0.016	0.014	2.016	0.016	0.014
	κ_2	1.01	1.015	0.005	0.004	1.015	0.005	0.004
	λ_1	4.5	4.493	0.007	0.029	4.493	0.007	0.029
	λ_2	50	50.095	0.095	15.336	50.095	0.095	15.336
n=1000	$\pi_1(x)$	–	–	0.013	0.001	–	0.286	0.109
	$\pi_2(x)$	–	–	0.013	0.001	–	0.286	0.109
	σ_e	7.746	7.745	0.001	0.006	7.745	0.001	0.006
	β_{11}	170	169.995	0.005	0.163	169.995	0.005	0.163
	β_{12}	88	88.006	0.006	0.065	88.006	0.006	0.065
	β_{21}	100	99.980	0.020	0.113	99.980	0.020	0.113
	β_{22}	1.2	1.207	0.007	0.035	1.207	0.007	0.035
	σ_b	7.071	7.051	0.020	0.039	7.051	0.020	0.039
	κ_1	2	2.010	0.010	0.007	2.010	0.010	0.007
	κ_2	1.01	1.014	0.004	0.002	1.014	0.004	0.002
	λ_1	4.5	4.504	0.004	0.015	4.504	0.004	0.015
	λ_2	50	50.071	0.071	7.059	50.070	0.070	7.058

Table S2: Bias and MSE Results for NN-JLCM $G = 2$ and JLCMM Parameter Estimates across Sample Sizes ($B = 1000$ simulations, 50% censoring)

			**NN-JLCM**			**JLCMM**		
Sample Size	Parameter	Truth	Estimate	AB	MSE	Estimate	AB	MSE
n=100	$\pi_1(x)$	–	–	0.014	0.007	–	0.283	0.118
	$\pi_2(x)$	–	–	0.014	0.007	–	0.283	0.118
	σ_e	7.746	7.734	0.012	0.060	7.734	0.012	0.060
	β_{11}	170	169.974	0.026	1.745	169.974	0.026	1.745
	β_{12}	88	87.976	0.024	0.682	87.975	0.025	0.681
	β_{21}	100	99.977	0.023	1.092	99.976	0.024	1.091
	β_{22}	1.2	1.213	0.013	0.381	1.213	0.013	0.381
	σ_b	7.071	6.985	0.086	0.387	6.985	0.086	0.387
	κ_1	2	2.116	0.116	0.122	2.114	0.114	0.120
	κ_2	1.01	1.052	0.042	0.032	1.051	0.041	0.032
	λ_1	4.5	4.499	0.001	0.215	4.500	0.000	0.214
	λ_2	50	51.426	1.426	153.588	51.423	1.423	153.608
n=500	$\pi_1(x)$	–	–	0.014	0.002	–	0.286	0.111
	$\pi_2(x)$	–	–	0.014	0.002	–	0.286	0.111
	σ_e	7.746	7.738	0.008	0.013	7.738	0.008	0.013
	β_{11}	170	170.031	0.031	0.381	170.030	0.030	0.380
	β_{12}	88	87.992	0.008	0.133	87.992	0.008	0.133
	β_{21}	100	100.010	0.010	0.220	100.010	0.010	0.220
	β_{22}	1.2	1.209	0.009	0.076	1.209	0.009	0.076
	σ_b	7.071	7.060	0.011	0.071	7.060	0.011	0.071
	κ_1	2	2.017	0.017	0.018	2.017	0.017	0.018
	κ_2	1.01	1.019	0.009	0.005	1.019	0.009	0.005
	λ_1	4.5	4.491	0.009	0.037	4.491	0.009	0.036
	λ_2	50	50.099	0.099	25.214	50.101	0.101	25.209
n=1000	$\pi_1(x)$	–	–	0.013	0.001	–	0.286	0.109
	$\pi_2(x)$	–	–	0.013	0.001	–	0.286	0.109
	σ_e	7.746	7.745	0.001	0.006	7.745	0.001	0.006
	β_{11}	170	169.995	0.005	0.163	169.995	0.005	0.162
	β_{12}	88	88.006	0.006	0.065	88.007	0.007	0.065
	β_{21}	100	99.980	0.020	0.113	99.981	0.019	0.113
	β_{22}	1.2	1.207	0.007	0.035	1.207	0.007	0.035
	σ_b	7.071	7.051	0.020	0.039	7.051	0.020	0.039
	κ_1	2	2.013	0.013	0.009	2.013	0.013	0.009
	κ_2	1.01	1.016	0.006	0.003	1.016	0.006	0.003
	λ_1	4.5	4.505	0.005	0.020	4.504	0.004	0.020
	λ_2	50	50.026	0.026	12.503	50.030	0.030	12.493

S1.2. Three-component model

Table S3: Bias and MSE Results for NN-JLCM $G = 3$ and JLCMM Parameter Estimates across Sample Sizes ($B = 1000$ simulations, 25% censoring)

			**NN-JLCM**			**JLCMM**		
Sample Size	Parameter	Truth	Estimate	AB	MSE	Estimate	AB	MSE
n=100	$\pi_1(x)$	–	–	0.004	0.058	–	0.459	0.283
	$\pi_2(x)$	–	–	0.021	0.061	–	0.243	0.141
	$\pi_3(x)$	–	–	0.023	0.114	–	0.435	0.349
	σ_e	1.000	0.996	0.004	0.002	0.996	0.004	0.002
	β_{11}	0.500	0.470	0.030	2.851	0.469	0.031	2.861
	β_{12}	2.000	1.982	0.018	0.169	2.014	0.014	1.239
	β_{21}	3.000	3.011	0.011	1.087	2.979	0.021	1.235
	β_{22}	2.000	2.012	0.012	1.227	2.021	0.021	0.568
	β_{31}	2.000	2.069	0.069	0.483	1.981	0.019	0.215
	β_{32}	1.000	0.988	0.012	0.073	0.983	0.017	0.092
	τ_{11}	3.000	2.954	0.046	0.078	2.941	0.059	0.099
	τ_{21}	1.000	0.987	0.013	0.041	0.985	0.015	0.045
	τ_{22}	1.732	1.701	0.031	0.024	1.695	0.037	0.030
	κ_1	2.000	2.148	0.148	0.208	2.150	0.150	0.221
	κ_2	5.000	6.388	1.388	10.090	6.431	1.431	16.189
	κ_3	10.000	10.334	0.334	1.655	10.501	0.501	2.851
	λ_1	4.500	4.467	0.033	0.269	4.474	0.026	0.300
	λ_2	50.000	49.907	0.093	12.020	48.976	1.024	22.356
	λ_3	25.000	24.979	0.021	0.137	24.989	0.011	0.181
	ω_1	2.500	2.461	0.039	0.121	2.420	0.080	0.138
	ω_2	1.500	1.493	0.007	0.047	1.655	0.155	0.139
n=500	$\pi_1(x)$	–	–	0.003	0.017	–	0.494	0.278
	$\pi_2(x)$	–	–	0.006	0.018	–	0.271	0.130
	$\pi_3(x)$	–	–	0.009	0.035	–	0.469	0.341
	σ_e	1.000	1.000	0.000	0.000	1.000	0.000	0.000
	β_{11}	0.500	0.520	0.020	0.522	0.520	0.020	0.525
	β_{12}	2.000	2.007	0.007	0.035	1.987	0.013	0.238
	β_{21}	3.000	3.015	0.015	0.196	3.002	0.002	0.216
	β_{22}	2.000	1.988	0.012	0.236	2.001	0.001	0.098
	β_{31}	2.000	2.008	0.008	0.088	2.004	0.004	0.040
	β_{32}	1.000	1.006	0.006	0.015	1.002	0.002	0.017
	τ_{11}	3.000	2.991	0.009	0.014	2.987	0.013	0.016
	τ_{21}	1.000	0.996	0.004	0.008	0.994	0.006	0.008
	τ_{22}	1.732	1.726	0.006	0.005	1.723	0.009	0.005
	κ_1	2.000	2.027	0.027	0.026	2.027	0.027	0.028
	κ_2	5.000	5.216	0.216	0.441	5.121	0.121	0.877
	κ_3	10.000	10.079	0.079	0.276	10.129	0.129	0.362
	λ_1	4.500	4.491	0.009	0.054	4.491	0.009	0.056
	λ_2	50.000	50.049	0.049	2.130	49.737	0.263	3.535
	λ_3	25.000	24.994	0.006	0.028	24.991	0.009	0.034
	ω_1	2.500	2.492	0.008	0.023	2.486	0.014	0.024
	ω_2	1.500	1.492	0.008	0.008	1.674	0.174	0.050
n=1000	$\pi_1(x)$	–	–	0.003	0.009	–	0.499	0.277
	$\pi_2(x)$	–	–	0.005	0.010	–	0.276	0.129
	$\pi_3(x)$	–	–	0.008	0.019	–	0.475	0.339
	σ_e	1.000	1.000	0.000	0.000	0.999	0.001	0.000
	β_{11}	0.500	0.501	0.001	0.239	0.536	0.036	0.238
	β_{12}	2.000	1.998	0.002	0.017	1.983	0.017	0.120
	β_{21}	3.000	2.987	0.013	0.106	2.969	0.031	0.114
	β_{22}	2.000	1.998	0.002	0.119	2.000	0.000	0.049
	β_{31}	2.000	2.000	0.000	0.043	1.992	0.008	0.019
	β_{32}	1.000	0.996	0.004	0.007	0.992	0.008	0.007
	τ_{11}	3.000	2.995	0.005	0.007	2.990	0.010	0.009
	τ_{21}	1.000	0.998	0.002	0.004	0.992	0.008	0.004
	τ_{22}	1.732	1.727	0.005	0.002	1.725	0.007	0.003
	κ_1	2.000	2.013	0.013	0.013	2.007	0.007	0.014
	κ_2	5.000	5.133	0.133	0.203	5.016	0.016	0.374
	κ_3	10.000	10.032	0.032	0.135	10.067	0.067	0.176
	λ_1	4.500	4.502	0.002	0.026	4.503	0.003	0.026
	λ_2	50.000	50.051	0.051	1.011	49.823	0.177	1.493
	λ_3	25.000	24.997	0.003	0.013	24.991	0.009	0.014
	ω_1	2.500	2.494	0.006	0.010	2.489	0.011	0.011
	ω_2	1.500	1.500	0.000	0.004	1.662	0.162	0.036

Table S4: Bias and MSE Results for NN-JLCM $G = 3$ and JLCMM Parameter Estimates across Sample Sizes ($B = 1000$ simulations, 50% censoring)

			**NN-JLCM**			**JLCMM**		
Sample Size	Parameter	Truth	Estimate	AB	MSE	Estimate	AB	MSE
n=100	$\pi_1(x)$	–	–	0.005	0.057	–	0.430	0.291
	$\pi_2(x)$	–	–	0.028	0.061	–	0.244	0.176
	$\pi_3(x)$	–	–	0.030	0.114	–	0.463	0.392
	σ_e	1	0.996	0.004	0.002	0.996	0.004	0.002
	β_{11}	0.5	0.487	0.013	2.902	0.484	0.016	2.930
	β_{12}	2	1.972	0.028	0.181	2.010	0.010	1.263
	β_{21}	3	3.044	0.044	1.181	2.936	0.064	1.697
	β_{22}	2	2.013	0.013	1.236	1.952	0.048	0.836
	β_{31}	2	2.087	0.087	0.560	1.941	0.059	0.935
	β_{32}	1	0.982	0.018	0.080	0.960	0.040	0.277
	τ_{11}	9	8.732	0.268	2.966	9.022	0.022	5.776
	τ_{21}	3	2.903	0.097	0.608	3.020	0.020	1.015
	τ_{22}	4	3.882	0.118	0.493	4.050	0.050	1.157
	κ_1	2	2.155	0.155	0.224	2.164	0.164	0.236
	κ_2	5	9.125	4.125	110.383	9.172	4.172	150.465
	κ_3	10	10.527	0.527	2.905	11.119	1.119	17.404
	λ_1	4.500	4.464	0.036	0.272	4.467	0.033	0.303
	λ_2	50	51.379	1.379	269.802	1.07×10^{61}	1.07×10^{61}	1.07×10^{62}
	λ_3	25	24.983	0.017	0.211	24.975	0.025	1.173
	ω_1	2.5	2.472	0.028	0.128	2.420	0.080	2.33×10^{12}
	ω_2	1.5	1.503	0.003	0.053	1.751	0.251	1.47×10^{19}
n=500	$\pi_1(x)$	–	–	0.003	0.017	–	0.483	0.277
	$\pi_2(x)$	–	–	0.007	0.018	–	0.273	0.140
	$\pi_3(x)$	–	–	0.010	0.035	–	0.479	0.354
	σ_e	1	1.000	0.000	0.000	1.001	0.001	0.000
	β_{11}	0.5	0.519	0.019	0.524	0.540	0.040	0.524
	β_{12}	2	2.005	0.005	0.036	2.006	0.006	0.249
	β_{21}	3	3.018	0.018	0.202	2.973	0.027	0.309
	β_{22}	2	1.989	0.011	0.238	1.971	0.029	0.145
	β_{31}	2	2.010	0.010	0.088	2.001	0.001	0.048
	β_{32}	1	1.004	0.004	0.015	0.995	0.005	0.022
	τ_{11}	9	8.939	0.061	0.531	8.956	0.044	1.046
	τ_{21}	3	2.976	0.024	0.112	2.990	0.010	0.169
	τ_{22}	4	3.974	0.026	0.100	3.993	0.007	0.213
	κ_1	2	2.028	0.028	0.028	2.025	0.025	0.028
	κ_2	5	5.488	0.488	1.495	5.378	0.378	2.844
	κ_3	10	10.112	0.112	0.438	10.199	0.199	0.608
	λ_1	4.5	4.491	0.009	0.055	4.489	0.011	0.061
	λ_2	50	49.937	0.063	5.696	48.853	1.147	18.252
	λ_3	25	24.993	0.007	0.043	24.987	0.013	0.062
	ω_1	2.5	2.495	0.005	0.023	2.493	0.007	0.028
	ω_2	1.5	1.494	0.006	0.009	1.701	0.201	0.074
n=1000	$\pi_1(x)$	–	–	0.003	0.009	–	0.490	0.276
	$\pi_2(x)$	–	–	0.005	0.010	–	0.278	0.138
	$\pi_3(x)$	–	–	0.008	0.019	–	0.482	0.350
	σ_e	1	1.000	0.000	0.000	0.999	0.001	0.000
	β_{11}	0.5	0.502	0.002	0.240	0.530	0.030	0.240
	β_{12}	2	1.998	0.002	0.017	1.983	0.017	0.121
	β_{21}	3	2.987	0.013	0.108	2.930	0.070	0.149
	β_{22}	2	1.999	0.001	0.119	1.961	0.039	0.077
	β_{31}	2	1.999	0.001	0.043	1.996	0.004	0.023
	β_{32}	1	0.995	0.005	0.007	0.994	0.006	0.009
	τ_{11}	9	8.980	0.020	0.259	8.996	0.004	0.503
	τ_{21}	3	2.993	0.007	0.057	2.992	0.008	0.091
	τ_{22}	4	3.985	0.015	0.044	3.989	0.011	0.094
	κ_1	2	2.015	0.015	0.014	2.010	0.010	0.015
	κ_2	5	5.243	0.243	0.542	5.035	0.035	1.131
	κ_3	10	10.040	0.040	0.200	10.104	0.104	0.305
	λ_1	4.5	4.498	0.002	0.026	4.498	0.002	0.027
	λ_2	50	50.049	0.049	2.790	49.165	0.835	12.711
	λ_3	25	24.994	0.006	0.020	24.982	0.018	0.023
	ω_1	2.5	2.494	0.006	0.011	2.486	0.014	0.012
	ω_2	1.5	1.500	0.000	0.004	1.680	0.180	0.050

Scientific notation used for extreme outlier values in JLCMM n=100 results

S1.3. Four component model

Table S5: Bias and MSE Results for NN-JLCM $G = 4$ and JLCMM Parameter Estimates across Sample Sizes ($B = 1000$ simulations, 25% censoring) Part I

			**NN-JLCM**			**JLCMM**		
Sample Size	Parameter	Truth	Estimate	AB	MSE	Estimate	AB	MSE
	$\pi_1(x)$	–	–	0.005	0.061	–	0.320	0.330
	$\pi_2(x)$	–	–	0.003	0.026	–	0.332	0.324
	$\pi_3(x)$	–	–	0.012	0.064	–	0.295	0.317
	$\pi_4(x)$	–	–	0.013	0.094	–	0.379	0.391
	σ_e	10	9.934	0.066	0.187	9.943	0.057	0.195
	β_{11}	66	66.239	0.239	17.034	68.706	2.706	100.760
	β_{12}	58	57.977	0.023	19.843	-10.451	68.451	5624.166
	β_{13}	65	64.965	0.035	15.873	-2.614	67.614	4720.495
	β_{21}	83	83.101	0.101	8.679	68.081	14.919	309.214
	β_{22}	14	13.700	0.300	44.994	-8.328	22.328	1394.848
	β_{23}	19	19.245	0.245	50.083	-3.115	22.115	628.215
	β_{31}	5	5.117	0.117	42.025	68.082	63.082	4060.925
	β_{32}	-66	-66.067	0.067	22.582	-5.699	60.301	4377.763
	β_{33}	-16	-15.921	0.079	5.981	-4.354	11.646	282.408
	β_{41}	-12	-12.058	0.058	6.657	68.081	80.081	6499.695
n=100	β_{42}	-3	-3.015	0.015	5.612	-8.328	5.328	924.703
	β_{43}	17	17.022	0.022	3.053	-3.115	20.115	543.754
	τ_{11}	15.362	14.809	0.554	2.954	15.932	0.570	3.339
	τ_{21}	-16.469	-15.583	0.885	11.293	-23.131	6.662	71.139
	τ_{31}	4.947	4.632	0.315	1.526	6.942	1.995	7.061
	τ_{22}	13.068	12.168	0.900	4.499	19.673	6.605	60.894
	τ_{32}	-4.555	-4.160	0.395	0.993	-6.373	1.818	6.167
	τ_{33}	0.882	0.368	0.514	0.532	0.685	0.197	0.411
	κ_1	30	33.017	3.017	71.350	26.636	3.364	148.447
	κ_2	40	46.437	6.437	264.271	27.864	12.136	229.260
	κ_3	43	48.602	5.602	188.865	28.098	14.902	322.966
	κ_4	39	41.194	2.194	44.186	27.712	11.288	218.739
	λ_1	76	75.917	0.083	0.401	†	†	†
	λ_2	98	97.917	0.083	0.604	†	†	†
	λ_3	89	88.960	0.040	0.334	8.52×10^{250}	8.52×10^{250}	†
	λ_4	83	82.974	0.026	0.160	†	†	†
	$\pi_1(x)$	–	–	0.003	0.017	–	0.334	0.328
	$\pi_2(x)$	–	–	0.002	0.007	–	0.367	0.352
	$\pi_3(x)$	–	–	0.003	0.017	–	0.337	0.332
	$\pi_4(x)$	–	–	0.005	0.027	–	0.388	0.371
	σ_e	10	9.999	0.001	0.035	9.996	0.004	0.036
	β_{11}	66	65.953	0.047	3.356	67.583	1.583	75.818
	β_{12}	58	58.024	0.024	3.700	-6.405	64.405	5016.353
	β_{13}	65	64.971	0.029	3.078	-4.188	69.188	4926.289
	β_{21}	83	83.013	0.013	1.835	67.700	15.300	306.144
	β_{22}	14	14.127	0.127	7.800	-6.228	20.228	1271.627
	β_{23}	19	18.943	0.057	9.235	-4.078	23.078	734.931
	β_{31}	5	5.008	0.008	7.920	71.116	66.116	4488.640
	β_{32}	-66	-66.016	0.016	4.273	-16.333	49.667	3625.239
	β_{33}	-16	-16.050	0.050	1.007	0.653	16.653	1822.338
	β_{41}	-12	-11.979	0.021	1.214	67.700	79.700	6424.050
n=500	β_{42}	-3	-2.997	0.003	1.028	-6.228	3.228	872.878
	β_{43}	17	17.007	0.007	0.539	-4.078	21.078	646.619
	τ_{11}	15.362	15.249	0.113	0.508	16.442	1.080	1.810
	τ_{21}	-16.469	-16.307	0.162	1.988	-24.323	7.854	69.616
	τ_{31}	4.947	4.893	0.054	0.266	7.248	2.300	6.054
	τ_{22}	13.068	12.853	0.215	0.707	20.902	7.834	67.330
	τ_{32}	-4.555	-4.460	0.095	0.157	-6.599	2.044	5.200
	τ_{33}	0.882	0.788	0.094	0.056	1.072	0.191	0.091
	κ_1	30	30.524	0.524	7.884	28.291	1.709	229.444
	κ_2	40	40.831	0.831	18.002	30.543	9.457	148.708
	κ_3	43	44.017	1.017	18.091	30.358	12.642	214.974
	κ_4	39	39.382	0.382	6.657	30.310	8.690	137.608
	λ_1	76	75.984	0.016	0.081	1.35×10^{176}	1.35×10^{176}	†
	λ_2	98	97.979	0.021	0.109	4.22×10^{13}	4.22×10^{13}	1.73×10^{27}
	λ_3	89	88.999	0.001	0.059	201.568	112.568	11724611.486
	λ_4	83	82.996	0.004	0.033	†	†	†

Table S6: Bias and MSE Results for NN-JLCM $G = 4$ and JLCMM Parameter Estimates across Sample Sizes ($B = 1000$ simulations, 25% censoring) Part II

			**NN-JLCM**			**JLCMM**		
Sample Size	Parameter	Truth	Estimate	AB	MSE	Estimate	AB	MSE
n=1000	$\pi_1(x)$	–	–	0.003	0.009	–	0.363	0.351
	$\pi_2(x)$	–	–	0.001	0.004	–	0.347	0.332
	$\pi_3(x)$	–	–	0.004	0.010	–	0.340	0.332
	$\pi_4(x)$	–	–	0.005	0.015	–	0.369	0.345
	σ_e	10	10.002	0.002	0.018	10.002	0.002	0.018
	β_{11}	66	66.023	0.023	1.610	67.164	1.164	66.607
	β_{12}	58	58.009	0.009	1.826	-4.428	62.428	4699.720
	β_{13}	65	65.024	0.024	1.640	-5.018	70.018	5036.173
	β_{21}	83	83.009	0.009	0.907	67.742	15.258	300.067
	β_{22}	14	13.972	0.028	4.011	-6.142	20.142	1225.277
	β_{23}	19	18.992	0.008	4.513	-4.465	23.465	688.909
	β_{31}	5	4.964	0.036	4.076	72.191	67.191	4749.704
	β_{32}	-66	-66.012	0.012	2.195	-23.665	42.335	2697.934
	β_{33}	-16	-15.991	0.009	0.521	2.539	18.539	630.179
	β_{41}	-12	-12.005	0.005	0.578	67.742	79.742	6426.094
	β_{42}	-3	-2.991	0.009	0.505	-6.142	3.142	829.455
	β_{43}	17	17.007	0.007	0.289	-4.465	21.465	599.048
	τ_{11}	15.362	15.300	0.062	0.247	16.530	1.168	1.718
	τ_{21}	-16.469	-16.361	0.108	0.980	-24.587	8.118	71.435
	τ_{31}	4.947	4.913	0.034	0.130	7.272	2.324	5.909
	τ_{22}	13.068	12.962	0.106	0.344	21.144	8.076	69.385
	τ_{32}	-4.555	-4.507	0.048	0.074	-6.546	1.991	4.571
	τ_{33}	0.882	0.843	0.039	0.019	1.122	0.240	0.080
	κ_1	30	30.257	0.257	3.347	27.033	2.967	238.764
	κ_2	40	40.590	0.590	8.915	30.002	9.998	143.171
	κ_3	43	43.500	0.500	8.338	30.046	12.954	216.089
	κ_4	39	39.181	0.181	3.414	29.618	9.382	133.398
	λ_1	76	75.995	0.005	0.041	†	†	†
	λ_2	98	97.996	0.004	0.056	132.325	34.325	1927845.145
	λ_3	89	89.006	0.006	0.031	6.38×10^{43}	6.38×10^{43}	†
	λ_4	83	82.994	0.006	0.016	1.64×10^{59}	1.64×10^{59}	†

† indicates infinite or extremely large values due to numerical instability

Table S7: Bias and MSE Results for NN-JLCM $G = 4$ and JLCMM Parameter Estimates across Sample Sizes ($B = 1000$ simulations, 50% censoring) Part I

			**NN-JLCM**			**JLCMM**		
Sample Size	Parameter	Truth	Estimate	AB	MSE	Estimate	AB	MSE
n=100	$\pi_1(x)$	–	–	0.006	0.059	–	0.314	0.327
	$\pi_2(x)$	–	–	0.004	0.025	–	0.337	0.330
	$\pi_3(x)$	–	–	0.012	0.063	–	0.285	0.307
	$\pi_4(x)$	–	–	0.013	0.092	–	0.379	0.390
	σ_e	10	9.935	0.065	0.185	9.942	0.058	0.189
	β_{11}	66	66.260	0.260	17.424	68.898	2.898	105.140
	β_{12}	58	57.950	0.050	20.320	-10.500	68.500	5620.568
	β_{13}	65	64.973	0.027	15.964	-2.838	67.838	4801.752
	β_{21}	83	83.100	0.100	8.889	67.626	15.374	328.118
	β_{22}	14	13.642	0.358	46.362	-7.191	21.191	1331.777
	β_{23}	19	19.289	0.289	51.207	-3.647	22.647	647.311
	β_{31}	5	5.139	0.139	42.453	67.865	62.865	4033.600
	β_{32}	-66	-66.105	0.105	23.243	-5.730	60.270	4379.729
	β_{33}	-16	-15.908	0.092	6.079	-4.265	11.735	375.412
	β_{41}	-12	-12.074	0.074	6.786	67.626	79.626	6431.997
	β_{42}	-3	-3.020	0.020	5.636	-7.191	4.191	900.277
	β_{43}	17	17.033	0.033	3.132	-3.647	20.647	560.723
	τ_{11}	15.362	14.803	0.560	2.977	15.972	0.609	3.469
	τ_{21}	-16.469	-15.558	0.911	11.342	-23.298	6.829	73.379
	τ_{31}	4.947	4.626	0.321	1.512	6.969	2.022	7.094
	τ_{22}	13.068	12.100	0.968	4.727	19.718	6.649	60.320
	τ_{32}	-4.555	-4.140	0.415	1.030	-6.340	1.785	5.834
	τ_{33}	0.882	0.367	0.514	0.523	0.673	0.208	0.452
	κ_1	30	34.332	4.332	130.072	25.611	4.389	161.729
	κ_2	40	51.265	11.265	666.603	27.078	12.922	262.321
	κ_3	43	52.240	9.240	487.211	27.333	15.667	345.316
	κ_4	39	42.109	3.109	77.701	27.289	11.711	241.042
	λ_1	76	75.880	0.120	0.585	†	†	†
	λ_2	98	98.384	0.384	112.335	3.95×10^{176}	3.95×10^{176}	†
	λ_3	89	88.901	0.099	0.522	4.43×10^{84}	4.43×10^{84}	1.87×10^{169}
	λ_4	83	82.974	0.026	0.237	†	†	†
n=500	$\pi_1(x)$	–	–	0.003	0.017	–	0.368	0.359
	$\pi_2(x)$	–	–	0.002	0.007	–	0.341	0.326
	$\pi_3(x)$	–	–	0.004	0.017	–	0.333	0.329
	$\pi_4(x)$	–	–	0.005	0.027	–	0.391	0.373
	σ_e	10	9.998	0.002	0.035	9.996	0.004	0.037
	β_{11}	66	65.941	0.059	3.417	67.424	1.424	78.174
	β_{12}	58	58.033	0.033	3.752	-5.722	63.722	4927.062
	β_{13}	65	64.974	0.026	3.085	-4.516	69.516	4978.381
	β_{21}	83	83.016	0.016	1.840	68.029	14.971	296.830
	β_{22}	14	14.145	0.145	7.950	-8.305	22.305	1379.579
	β_{23}	19	18.939	0.061	9.303	-3.311	22.311	645.523
	β_{31}	5	5.001	0.001	7.956	71.032	66.032	4471.392
	β_{32}	-66	-66.027	0.027	4.283	-15.621	50.379	3355.572
	β_{33}	-16	-16.057	0.057	1.027	-0.682	15.318	386.568
	β_{41}	-12	-11.976	0.024	1.221	68.029	80.029	6477.337
	β_{42}	-3	-2.995	0.005	1.032	-8.305	5.305	910.195
	β_{43}	17	17.010	0.010	0.541	-3.311	20.311	560.279
	τ_{11}	15.362	15.250	0.113	0.506	16.446	1.084	1.848
	τ_{21}	-16.469	-16.305	0.164	1.997	-24.340	7.871	70.344
	τ_{31}	4.947	4.892	0.055	0.268	7.253	2.306	6.097
	τ_{22}	13.068	12.846	0.222	0.712	20.908	7.840	67.557
	τ_{32}	-4.555	-4.458	0.097	0.157	-6.610	2.055	5.298
	τ_{33}	0.882	0.785	0.097	0.058	1.069	0.188	0.094
	κ_1	30	30.700	0.700	10.852	28.676	1.324	235.295
	κ_2	40	41.353	1.353	30.551	30.458	9.542	154.235
	κ_3	43	44.512	1.512	30.428	30.191	12.809	232.850
	κ_4	39	39.566	0.566	10.216	30.213	8.787	140.535
	λ_1	76	75.980	0.020	0.115	†	†	†
	λ_2	98	97.958	0.042	0.187	†	†	†
	λ_3	89	88.996	0.004	0.086	6.44×10^{209}	6.44×10^{209}	†
	λ_4	83	83.001	0.001	0.047	8.30×10^{15}	8.30×10^{15}	6.52×10^{31}

Table S8: Bias and MSE Results for NN-JLCM $G = 4$ and JLCMM Parameter Estimates across Sample Sizes ($B = 1000$ simulations, 50% censoring) Part II

			**NN-JLCM**			**JLCMM**		
Sample Size	Parameter	Truth	Estimate	AB	MSE	Estimate	AB	MSE
n=1000	$\pi_1(x)$	–	–	0.003	0.009	–	0.333	0.324
	$\pi_2(x)$	–	–	0.002	0.004	–	0.348	0.334
	$\pi_3(x)$	–	–	0.004	0.010	–	0.335	0.329
	$\pi_4(x)$	–	–	0.005	0.015	–	0.368	0.346
	σ_e	10	10.002	0.002	0.018	10.002	0.002	0.019
	β_{11}	66	66.021	0.021	1.625	67.826	1.826	71.801
	β_{12}	58	58.003	0.003	1.856	-6.974	64.974	5049.711
	β_{13}	65	65.015	0.015	1.649	-4.025	69.025	4901.130
	β_{21}	83	83.009	0.009	0.912	66.860	16.140	323.527
	β_{22}	14	13.987	0.013	4.034	-2.779	16.779	1044.339
	β_{23}	19	19.002	0.002	4.565	-5.879	24.879	746.771
	β_{31}	5	4.977	0.023	4.095	72.856	67.856	4738.016
	β_{32}	-66	-66.011	0.011	2.181	-23.929	42.071	2633.183
	β_{33}	-16	-15.997	0.003	0.526	2.494	18.494	508.134
	β_{41}	-12	-12.006	0.006	0.585	66.860	78.860	6281.963
	β_{42}	-3	-2.995	0.005	0.509	-2.779	0.221	762.869
	β_{43}	17	17.007	0.007	0.287	-5.879	22.879	651.255
	τ_{11}	15.362	15.299	0.063	0.250	16.524	1.161	1.726
	τ_{21}	-16.469	-16.354	0.115	0.992	-24.600	8.131	71.656
	τ_{31}	4.947	4.911	0.036	0.131	7.283	2.336	5.942
	τ_{22}	13.068	12.954	0.114	0.350	21.181	8.112	69.668
	τ_{32}	-4.555	-4.505	0.050	0.076	-6.585	2.030	4.788
	τ_{33}	0.882	0.840	0.041	0.019	1.124	0.242	0.083
	κ_1	30	30.384	0.384	4.745	26.695	3.305	236.128
	κ_2	40	40.877	0.877	15.152	29.797	10.203	154.827
	κ_3	43	43.722	0.722	13.641	30.201	12.799	218.778
	κ_4	39	39.313	0.313	4.806	30.136	8.864	125.129
	λ_1	76	75.987	0.013	0.056	†	†	†
	λ_2	98	97.988	0.012	0.090	6383.632	6285.632	3.52×10^{10}
	λ_3	89	89.004	0.004	0.048	5785849.680	5785760.680	2.71×10^{16}
	λ_4	83	82.994	0.006	0.023	198509.765	198426.765	3.55×10^{13}

† indicates infinite or extremely large values due to numerical instability
